# Distal electrical stimulation enhances neuromuscular reinnervation and satellite cell differentiation for functional recovery

**DOI:** 10.1186/s13287-025-04459-3

**Published:** 2025-06-23

**Authors:** Chun-Wei Lin, Szu-Han Chen, Siao Muk Cheng, Tzu-Chun Chung, Wentai Liu, Daw-Yang Hwang, Song Li, Sheng-Che Lin, Yuan-Yu Hsueh

**Affiliations:** 1 School of Medicine, College of Medicine, National Cheng Kung University, Tainan, 701 Taiwan; 2 Division of Plastic and Reconstructive Surgery, Department of Surgery, College of Medicine, National Cheng Kung University Hospital, National Cheng Kung University, Tainan, 701 Taiwan; 3 Center of Transformative Bioelectronic Medicine, National Cheng Kung University, Tainan, 701 Taiwan; 4 Institute of Clinical Medicine, College of Medicine, National Cheng Kung University, Tainan, 701 Taiwan; 5 National Institute of Cancer Research, National Health Research Institutes, Tainan, 704 Taiwan; 6 Department of Orthopedics, E-Da Hospital, Kaohsiung, 824 Taiwan; 7 Department of Bioengineering, University of California, Los Angeles, CA 90095 USA; 8 Department of Electrical and Computer Engineering, UCLA, Los Angeles, CA 90095 USA; 9 California Nanosystems Institute, UCLA, Los Angeles, CA 90095 USA; 10 Brain Research Institute, UCLA, Los Angeles, CA 90095 USA; 11 Division of Plastic Surgery, Department of Surgery, An-Nan Hospital, China Medical University, Tainan, 709 Taiwan; 12 Department of Physiology, College of Medicine, National Cheng Kung University, Tainan, 701 Taiwan

**Keywords:** Electrical stimulation, Neuromuscular regeneration, Satellite cell, Denervation muscle injury, Peripheral nerve regeneration

## Abstract

**Background:**

Peripheral nerve injuries lead to significant motor deficits, with limited treatment options for full functional recovery. Distal electrical stimulation (E-stim) has shown promise in promoting neuromuscular reinnervation, though its mechanisms are not yet fully understood. This study aims to investigate the regulatory effects of distal E-stim on neuromuscular junction (NMJ) reinnervation and Satellite cell activity in denervated muscle injury.

**Methods:**

Using a sciatic nerve critical gap model in Sprague-Dawley rats (8-week-old, random sex), we applied distal E-stim and assessed neuromuscular and functional recovery through histological, biochemical, and functional evaluations over six weeks. The Sciatic Function Index (SFI) was measured at baseline and at subsequent time points post-injury. We quantified muscle mass, NMJ morphology, and neurotransmitter levels (acetylcholine and acetylcholinesterase), and analyzed muscle fiber electrophysiology using single-muscle electromyography to assess denervated muscle autoelectricity. Additionally, single-cell RNA sequencing was performed to examine gene expression in Satellite cells.

**Results:**

Distal E-stim significantly enhanced neuromuscular reinnervation, as evidenced by improved SFI scores, increased muscle mass, and reduced muscle atrophy. Histological analysis showed larger muscle fiber cross-sectional areas and enhanced NMJ structure. Elevated levels of acetylcholine and acetylcholinesterase, along with reduced fibrillation potentials in muscle fibers, further indicated preserved NMJ function. Single-cell RNA sequencing revealed upregulation of genes associated with muscle differentiation and angiogenesis in Satellite cell clusters, suggesting that distal E-stim fosters a regenerative environment.

**Conclusions:**

Our findings demonstrate that distal E-stim promotes functional recovery through NMJ preservation and Satellite cell differentiation, offering novel insights into molecular mechanisms that may enhance electroceutical therapies for peripheral nerve injuries. Further research could optimize E-stim protocols to maximize clinical benefits for patients with neuromuscular impairments.

**Graphical abstract:**

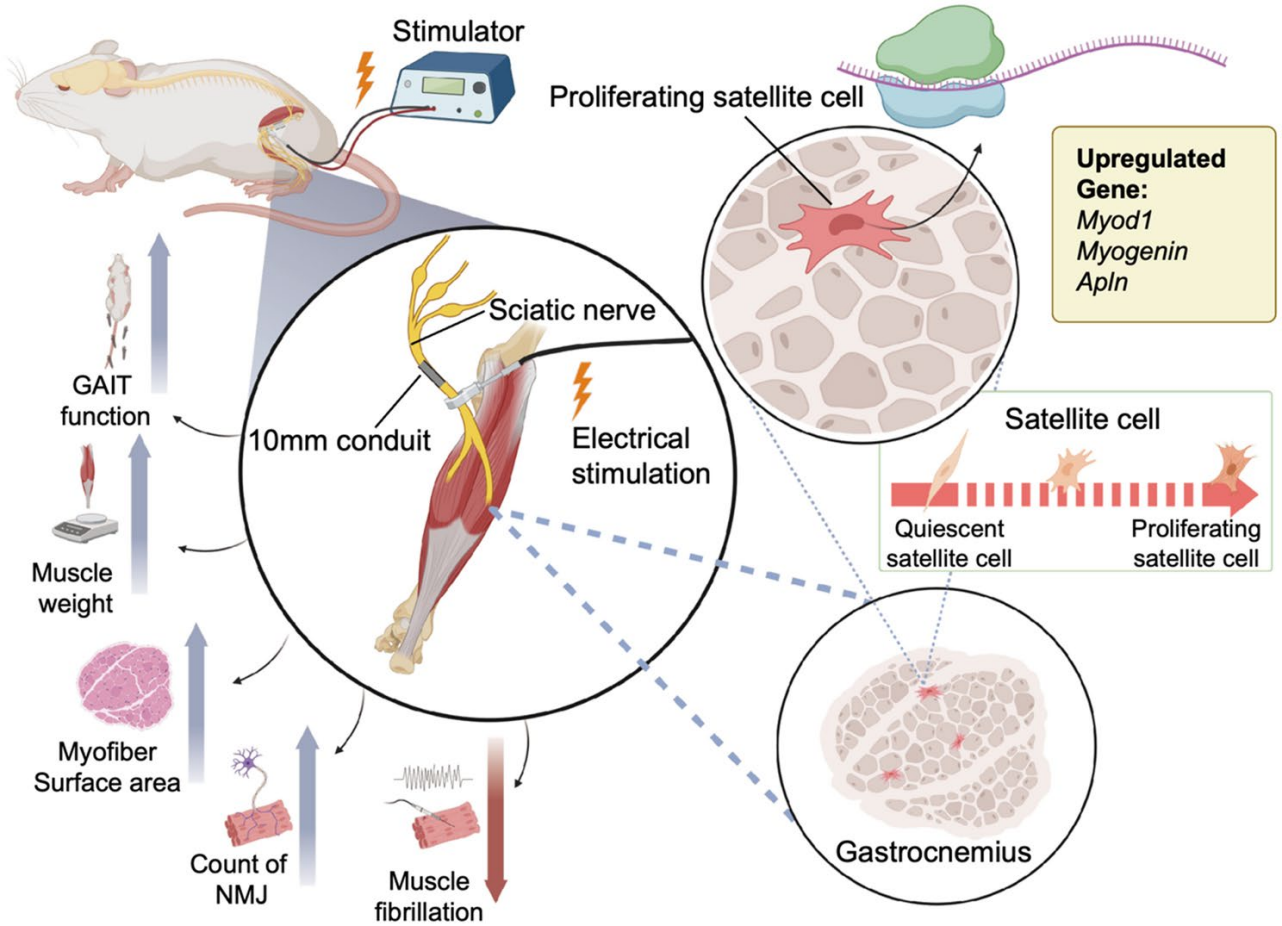

**Supplementary Information:**

The online version contains supplementary material available at 10.1186/s13287-025-04459-3.

## Background

Peripheral nerve injuries significantly impact individuals, affecting their quality of life, daily functioning, and emotional well-being. These injuries can lead to extensive sensory and motor functional loss, such as persistent pain, hyperesthesia, and physical disabilities [1]. Patients with severe nerve injuries, like brachial plexus injury or mangled extremities, often experience a reduced quality of life, increased pain levels, and heightened stress [2]. Moreover, peripheral nerve trauma can result in long-lasting functional disabilities, especially in younger individuals, necessitating tailored rehabilitation to optimize outcomes [3]. Despite advancements in treatment strategies, achieving full functional recovery after severe nerve injuries remains challenging. This underscores the need for further research and innovative approaches, such as electrical stimulation, to enhance nerve regeneration and functional neuromuscular recovery [4].

Neuromuscular regeneration is a complex process essential for functional recovery following nerve injuries or motor neuron diseases. The establishment of a regenerative niche at the neuromuscular junction (NMJ) is critical for successful regeneration, ensuring proper axon arrival and postsynaptic domain resilience [5, 6]. Upon the arrival of a motor nerve action potential, calcium influx triggers the release of acetylcholine (ACh) into the synaptic gap, which is recycled back to presynapse by acetylcholinesterase (AChE). This ACh binds to receptors on the muscle fiber surface, initiating the endplate potential, which leads to muscle action potential and contraction [7]. The intricate structures of the NMJ convert nerve impulses into muscle action potentials necessary for contraction. Additionally, their plasticity allows for structural and functional adaptations throughout life and under pathological conditions, maintaining consistent activation and adjusting for various motor unit types. Studies have shown that reversible nerve damage can have long-lasting effects on NMJ components, influencing functional recovery and synaptic adaptations [8]. Understanding the cellular and molecular responses involved in NMJ regeneration is crucial for developing effective strategies to restore motor function in conditions affecting peripheral NMJ innervation [9].

Satellite cells play a crucial role in NMJ regeneration by contributing to muscle fiber repair and regeneration processes [10, 11]. These myogenic stem cells are activated upon muscle injury, undergoing symmetric divisions to generate new stem cells and myoblasts. These myoblasts then differentiate into muscle cells to rebuild the muscle fiber, supporting skeletal muscle regeneration [12]. Satellite cells communicate within their niche to mediate muscle remodeling in response to exercise, regulating various cell types through secretory signaling and cell-cell contact, ultimately contributing to muscle adaptation and repair [13]. Additionally, Satellite cells provide new myoblasts during muscle growth and regeneration, making them integral to restoring NMJ function.

Electrical stimulation (E-stim) has been shown to enhance peripheral nerve regeneration. It promotes axon growth, accelerates sensorimotor recovery, and fosters muscle reinnervation by increasing the expression of protective proteins and neurotrophic factors [14]. Various types of E-stim, such as neuromuscular, transcutaneous, and functional electrical stimulation, have been used to modulate sensory feedback, reduce neuralgia, and prevent muscle atrophy [15]. Studies have demonstrated that brief low-frequency proximal electrical stimulation effectively promotes nerve regeneration, enhances motor function, accelerates Wallerian degeneration, upregulates the expression of neurotrophic factors, and speeds up nerve regeneration after injury [16–18]. Clinical trials suggest that low-intensity E-stim post-surgery can positively impact sensory function recovery in patients with digital nerve injuries, carpal tunnel syndrome, tardy ulnar palsy, and brachial plexus injuries [14, 19]. The efficacy of E-stim in peripheral nerve injuries is evident in improved functional recovery, making it a valuable non-pharmacological intervention for enhancing peripheral nerve regeneration [18, 20].

However, there is limited evidence supporting the use of E-stim to enhance NMJ regeneration. Recently, we demonstrated that direct E-stim applied distal to the nerve injury site facilitates functional muscle regeneration by direct promoting NMJ regeneration [21]. This beneficial effect with its underlying molecular mechanism differs from the previously established proximal E-stim [18] that induces intracellular calcium influx in neuronal cells, leading to the release of neurotrophic factors and subsequent robust axon sprouting and regeneration. In this research, we aim to further explore the underlying molecular mechanisms of distal E-stim on neuromuscular functional regulation, with a particular focus on the regenerative NMJ niche and Satellite cell function within denervated muscle, using single-cell RNA sequencing (scRNA-seq) analysis and functional evaluation.

## Methods

### Animals and stimulation protocol

The work has been reported in line with the ARRIVE guidelines 2.0. This study aimed to investigate the effects of distal nerve E-stim on neuromuscular reinnervation and functional recovery following peripheral nerve injury. The animals were housed at the National Cheng Kung University Experimental Animal Center and were given at least one week to acclimate before the nerve injury experiments. We utilized a rat sciatic nerve critical gap (10 mm) model to simulate severe nerve injury, as in our previous publication [22]. Adult Sprague-Dawley rats (8-week-old, random sex, weight 180–220 g, *n* = 45) were randomly assigned into three groups: Naive (*n* = 15), Control (*n* = 15), and E-stim (*n* = 15) by simple randomization method. The Naive group had no surgical intervention and served as the baseline. The Control group underwent left sciatic nerve transection and repair using a 10-mm conduit to create a critical nerve gap without E-stim. The E-stim group received the same surgical procedure as the Control group, followed by a single session of distal electrical stimulation (20 Hz, 30 min, 200 µsec, 0.1–0.4 mA, balanced biphasic square wave) [21]. At the end of evaluation, the animals were euthanized by overdose of 5% Isoflurane, and specimens were collected only after confirming the animals without breathing and heartbeat. All animal care and procedures were approved by the Institutional Animal Care and Use Committee of National Cheng Kung University under protocol no. 108,237, including pain control, observation of adverse events, and humane endpoints during the entire experiment.

### Dynamic gait analysis

For the assessment of motor nerve recovery, Motion Gait Analysis was conducted as previously described with minor modifications [22]. All animals were handled gently and tested in a quiet environment to minimize stress. Prior to testing, the rats underwent conditioning trials on a 6 × 80 cm walking track to familiarize them with the testing environment and procedure. Image processing was performed using MATLAB software. The print length (PL), toe spread (TS), and intermediary toe spread (IT) were obtained according to the following calculation formula:

$$\eqalign{& {\rm{SFI}}\; = \;38.3\;({\rm{EPL}} - {\rm{NPL}})/{\rm{NPL}}\; + \;109.5\;({\rm{ETS}}\; - \; \cr & {\rm{NTS}})\;/\;{\rm{NTS}}\; + \;13.3\;({\rm{EIT}} - {\rm{NIT}})/{\rm{NIT}}\; - 8.89 \cr} $$

In general, the maximal value was adopted for each measurement to ensure consistency and accuracy. The data was analyzed using Rat Motion Gait Software. The Sciatic Function Index (SFI), an indicator of the degree of nerve dysfunction, ranged from 0 to -100, with 0 corresponding to normal function and − 100 indicating complete dysfunction. For each walking track, three footprints were analyzed by a single observer to ensure consistency and reliability. The average of these measurements was used for SFI calculations.

### Hematoxylin and eosin stain

The paraformaldehyde-fixed muscle segments were dehydrated and then embedded in paraffin. The muscle tissues were sectioned transversely at 4–5 μm. Hematoxylin and eosin (H&E) staining was then applied to observe tissue morphology.

### NMJ staining and immunofluorescence

The muscle and nerve tissue was embedded in OCT (Tissue-Tek^®^, Sakura Finetek Inc, Torrence, CA, USA) and deep-frozen until use.The area of NMJ specializations was assessed using longitudinal sections of the diaphragm muscle 35 μm and the nerve Sect. 8 μm. The primary antibodies used were monoclonal anti-Neurofilament 200 (N0142, 1:200; Sigma-Aldrich) overnight at 4 °C. After washing three times with 1XPBS, sections were incubated with fluorescene isothiocyanate-conjugated Goat anti-Mouse IgG (GTX213111-04, 1∶200; Gene Tex) and Alexa594-α-bungarotoxin (B13423, 1:200; Invitrogen™) for 2 h at room temperature. Image stacks for maximum-intensity Z-projection images were collected by using confocal laser scanning microscopes (FV3000, Olympus, Tokyo, Japan), and imagej software 1.54.3 was used to measure the numbers of the NMJ of each group. Immunofluorescent staining was performed to detect specific expression patterns exhibited by proteins. The primary antibodies used were anti-Neurofilament 200 (NF200, N0142, 1:200; Sigma-Aldrich); (s100beta, ab52642; 1:200; abcam); (IL-1β, GTX74034, 1:200; Genetex); (TNF-α, 1:200; GTX110520; Genetex ) overnight at 4 °C. After washing three times with 1XPBS, sections were incubated with fluorescene isothiocyanate TRITC-conjugated Goat anti-Mouse IgG (GTX213111-05, 1∶200; Gene Tex) and fluorescene isothiocyanate-conjugated Goat anti- rabbit IgG and mounted in Mounting Medium with DAPI (ab104139 abcam). Slices were visualized and photographed under fluorescence microscopy (BX53, Olympus, Tokyo, Japan).

### Single fiber electromyography

Rats were anesthetized using 3% Isoflurane administered via inhalation and positioned on their right side on an electrically heated pad within a warm room. To ensure consistent body temperature, their exposed hindquarters and left leg were covered with a jacket composed of a parallel array of silicone rubber tubes, through which water maintained at 37 °C was circulated. Recordings were initiated only when the rectal temperature and skin temperature over the thigh and ankle stabilized at 37 ± 0.5 °C. If the animals’ temperatures were below this threshold at the start of the experiment, they were warmed accordingly. For the electromyography recording setup [23], disposable acupuncture needles were inserted into the belly and tendon of each muscle. A recording concentric needle electrode (Ambu^®^ Neuroline™ Concentric, 25 mm x 0.30 mm [30G], recording area 0.02mm^2^, Ambu A/S, Dk-2750 Ballerup-Denmark’s) was placed in the middle of the gastrocnemius muscle, a reference needle electrode was positioned near the Achilles tendon, and a ground electrode was affixed to the tail. Single Fiber Electromyography (SFEMG) was employed to monitor muscle fiber electrophysiological properties at post-injury day 6, 9, 14. The frequency of fibrillation was calculated by measuring multiple sites within a muscle and determining the mean number of fibrillation potentials with amplitudes greater than 30 µV. The Nihon Kohden Neuropack^®^ X1 MEB-2300 EMG/NCV/EP Measuring Desktop System (Japan Inc.) was utilized for both testing and data acquisition.

### Biochemical analyses

The total protein of each group was homogenized in 1X PBS. The homogenate was centrifuged at 8,000 g for 10 min at 4 ℃. The protein concentration was determined with the Pierce™ BCA Protein Assay Kit (Cat. #23225, Thermo Fisher Scientific^®^, Waltham, MA, USA) and subjected to analysis on a AchE ELISA Kit (Cat. No: OKEH03313, Aviva Systems Biology) and Ach ELISA Kit (Cat. No: E-EL-0081, Elasscience) according to the manufacturer’s reagents and protocols. The optical density of each well were determined with a micro-plate reader (SpectraMax i3x, Molecular Devices) set to 450 nm.

### RNA isolation, quantitative PCR

The total RNA was extracted from the muscle samples of each group using TRIzol reagent (Sigma-Aldrich, St. Louis, MO, USA) according to the manufacturer’s instructions. The total RNA was precipitated with 2-propanol, washed twice with 75% ethanol, and resuspended in diethylpyrocarbonate-treated water. The total RNA concentration was determined by measuring the optical density values of the samples at 260 nm. To prepare first-strand cDNA, mRNA was reversetranscribed with the reverse transcriptase enzyme (ImProm-II™ Reverse Transcriptase, Promega, Madison, WI, USA) in a reaction mixture (20 µL in total). Primers were designed for α-AchR, Atrogin-1, N-CAM, Musk and glyceraldehyde 3-phosphate dehydrogenase (GAPDH). GAPDH was used as a reference gene. Quantitative PCR was performed to analyze the mRNA levels, using the SYBR PCR Master Mix (GoTaq Green Master Mix, Promega, Madison, WI, USA) and StepOnePlus™ System (Thermo Fisher Scientific, Waltham, MA, USA). The quantitative PCR conditions were as follows: 10 min at 95℃, followed by 40 cycles of 15 s at 95℃ and 30 s at 60℃. The 2^–ΔCT^ method was used to analyze the quantitative PCR data.

### Muscle cell isolation and sample collection

Single-cell suspensions were obtained from rat skeletal muscle using the Skeletal Muscle Dissociation Kit (MACS 130-098-305) and subsequently incubated with 1 ml RBC lysis buffer for 2 min in room temperature. Cells were then centrifuged at 500G for 5 min and resuspended in Dulbecco’s modified Eagle’s medium (Hyclone) supplemented with 10% fetal bovine serum (Hyclone). An aliquot of the single cell suspension was stained with the trypan blue (Invitrogen) and measured cell viability and number with TC20™ automated cell counted (Bio-rad) to ensure cell viability was greater than 80%.

### Single-cell RNA sequencing library preparation

scRNA-seq libraries were prepared with Chromium Single cell 5’ Reagent Kits v2 (10X Genomics, USA) following the manufacturer’s protocol. In brief, 10,000 cells which mixed with reverse transcription reagent, gel beads and partitioning oil were loaded into a Chromium Next GEM Chip K. Then, Chip K was subsequently loaded into a Chromium Controller to generate gel bead-in-emulsions and follow by reverse transcripts (53 °C for 45 min; 85 °C for 5 min) to produced cDNA. cDNA were then cleanup with Dynabeads and followed by cDNA amplification [98 °C for 45 s; (98 °C for 20 s, 63 °C for 30 s and 72 °C for 1 min) × 11 cycles; 72 °C for 1 min]. The amplified cDNA was then fragmented, ligated with adapter and sample index, and performed double sided selection with SPRI beads (Beckman) according to manufacturer’s protocol. Finally, the libraries were quantified with Qubit dsDNA HS assay kit (ThermoFisher), and the libraries average fragment size were measured with 4150 Tapestation system (Agilent). Finally, libraries were sequenced on an Illumina NextSeq 2000 platform. A total of 2 × 150 bp paired-end runs of Illumina NextSeq 2000 were performed according to manufacturer’s protocol.

### Sequencing data processing and analysis

Sequencing data were imported to Cellranger (version 6.0.1) for further analysis. We used the cellranger mkfastq function to demultiplex base-call files into FASTQ files, followed by the cellranger count function to perform alignment, filtering, barcode counting, and Unique Molecular Identifier counting. Next, we used the Seurat package (version 4.0.3) in R for further analysis. To remove low-quality and doublet cells, we excluded cells with fewer than 600 genes and more than 3000 genes, as well as those with mitochondrial gene percentages higher than 40%, which could indicate potential apoptotic cells. We integrated our samples using the standard integration protocol described in the Seurat package to correct batch effects between datasets. Individual datasets were normalized using the NormalizeData function, and 2,000 highly variable genes were selected using the FindVariableFeatures function under default parameters. We then used the FindIntegrationAnchors function to identify integration anchors between datasets, followed by the IntegrateData function to integrate all datasets. The dimensionality reduction plot (DimPlot) splited by condition showed the absent of discrete cluster within conditions, indicating that no batch effect existing, and the experimental condition response for the observed differences instead of artifacts (Fig. S1). Then the function Findcluster was applied to cluster the data. The optimal resolution for clustering was determined by Clusetree package in R. Differentially expressed genes (DEGs) in each cluster were identified with log2-fold change > 0.5 with p value < 0.05 in Seurat FindAllMarkers function. Cell types annotation was assisted by ScCatch package in R, and annotated by known housekeeping gene for each clustered cell type.

### Gene ontology (GO) analysis

GO enrichment analysis was done using ClusterProfiler package in R, comparing DEGs in E-stim condition versus Control, and employing the annotation database org.Rn.eg.db. The results of DEGs was used as input and the following options: ont ="ALL”, keyType = “SYMBOL”, nPerm = 10000, minGSSize = 10, maxGSSize = 200, pvalueCutoff = 0.05, verbose = TRUE, OrgDb = org.Rn.eg.db, pAdjustMethod = “none”). Terms with adjusted p values < 0.05 were considered significant. The GO terms of GSEA were clustered with the ‘binary cut’ algorithm using the R package simplifyEnrichment to further analyze the GSEA results. In the process of ‘binary cut’ clustering, difference score, cluster numbers and other parameters were set following the R package documentation. Heatmaps with word cloud annotations were generated to summarize the functions of each enriched GO cluster. Dotplots and the Centplots aimed to express the upregulation level of interested genes in experimental groups, and show the network of genes and GO term respectively were generated using ggplot2 (3.3.5).

### Statistical analysis

All statistical analyses were performed using GraphPad Prism v8, with data presented as means ± standard derivation and significance set at *P* < 0.05. One-way ANOVA followed by Tukey’s post hoc test was used for comparisons involving more than two groups, while unpaired t-tests were used for two-group comparisons, and the Wilcoxon signed-rank test was applied for paired comparisons. scRNA-seq data were analyzed to identify differentially expressed genes and pathways in Satellite cells three days post-injury, with statistical significance determined using appropriate bioinformatics tools. Specific statistical tests used are reported in each figure legend. The project leader, Chun-Wei Lin was the only one who was aware of the group allocation throughout the experiments.

## Results

### Distal nerve electrical stimulation facilitates functional recoveries and muscle regeneration

**Fig. 1:**
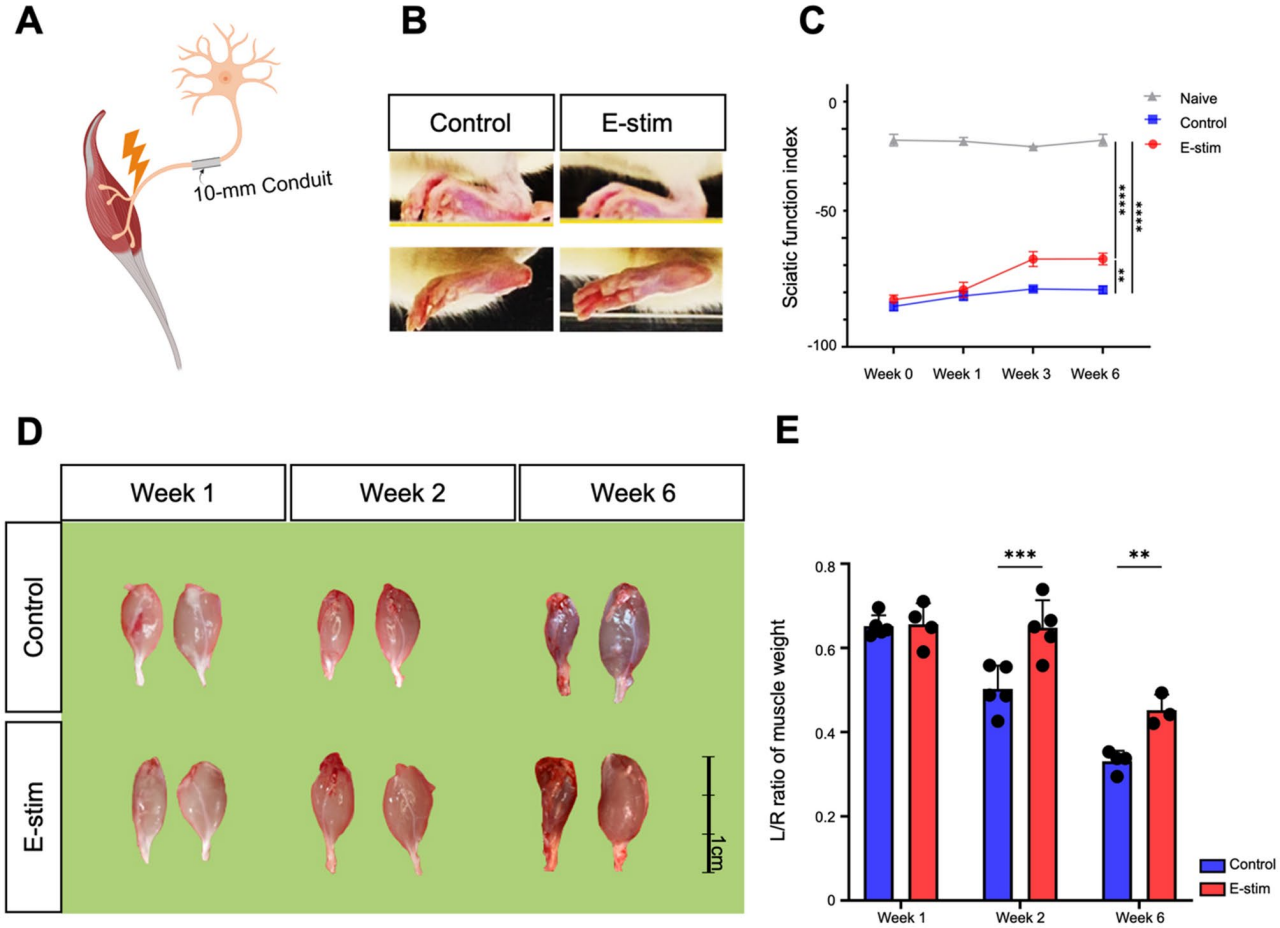
Distal electric stimulation improves functional recoveries. (**A**) Schematic illustration depicting electric stimulation applied to the distal stump of the nerve gap in a SD rat model. The “Naive” group is defined as having both hind legs intact. In the “Control” group, the left side underwent transection surgery with ligation using a 10 mm conduit, while the right side remained intact. In the “E-stim” group, the left side underwent nerve transection surgery with ligation using a 10 mm conduit, followed by a single instance of electrical stimulation; the right side remained intact. (**B**,** C**) A higher level of toe spreading was observed in the E-stim group. Sciatic function index data obtain at postoperative weeks1, 2 and 6. Each group comprised 3 independent animals, compared to the Naïve group. (*n* = 3 per timepoint. Data was presented with mean standard deviation. * indicated *p* < 0.05; ** indicated *p* < 0.01, *** indicated *p* < 0.001). **(D**,** E**) Gastrocnemius muscles were harvested from both hind legs of the Control group and the E-stim group at postoperative weeks 1, 2, and 6 following nerve transection. Muscle weight (MW) preservation rate was calculated by normalizing the left gastrocnemius muscle weight to that of the contralateral muscle. Scale bar = 1 cm. (*n* = 5 per timepoint. Data was presented with mean standard deviation. * indicated *p* < 0.05; ** indicated *p* < 0.01, *** indicated *p* < 0.001)

We investigated the therapeutic effect of distal nerve E-stim on nerve regeneration and muscle recovery using a 10-mm conduit model for sciatic nerve injury. The study compared outcomes between control and E-stim groups over six weeks, assessing functional and morphological recoveries. A schematic representation (Fig. 1A) illustrates direct distal nerve E-stim in the 10-mm conduit model used for nerve regeneration experiments. Morphological examination of the injured hindlimb (Fig. 1B) in the E-stim groups revealed alleviation of claw tow deformity as compared to the control group, indicating sciatic function recovery. Functional recovery was further assessed using the SFI (Fig. 1C), measured at baseline (week 0) and subsequent time points (weeks 1, 3, and 6). The E-stim group showed a significant improvement in SFI compared to the control group. At week 6, the E-stim group had a higher SFI, indicating better functional recovery of the sciatic nerve (E-stim: -59.7 ± 3.4; Control: -74.7 ± 1.9). Gross examination of muscle mass (Fig. 1D) at weeks 1, 2, and 6 post-injury showed a progressive difference between the control and E-stim groups. Muscles in the E-stim group appeared larger compared to the control group, particularly at week 6. This observation was quantified by measuring the left/right (L/R) ratio of muscle weight (Fig. 1E). The E-stim group demonstrated significantly higher L/R muscle weight ratios at weeks 2 and 6 compared to the control group (Week 2: E-stim: 0.65 ± 0.07, Control: 0.50 ± 0.06; *p* < 0.001; Week 6: E-stim: 0.45 ± 0.04, Control: 0.03 ± 0.02; *p* < 0.01).

To further investigate the effect of distal E-stim, we assessed the proximal nerve stump using immunofluorescent staining at week 6. The percentage of S100β+/NF200 + cells in the E-stim group did not show significant differences compared to the other two groups, indicating no significant axon sprouting in the proximal nerve stump (Fig. S2). Additionally, the levels of IL-1β+ (Fig. S3) and TNF-α+ (Fig. S4) cells were not significantly different in the E-stim group compared to controls, suggesting that distal E-stim does not contribute to neuroinflammation in the proximal nerve stump. Taken together, the application of distal E-stim significantly enhances the recovery of sciatic nerve function and muscle mass regeneration post-injury, without contributing to axon sprouting and neuroinflammation in the proximal nerve stump.

### Distal nerve electrical stimulation impedes denervated muscle atrophy

**Fig. 2:**
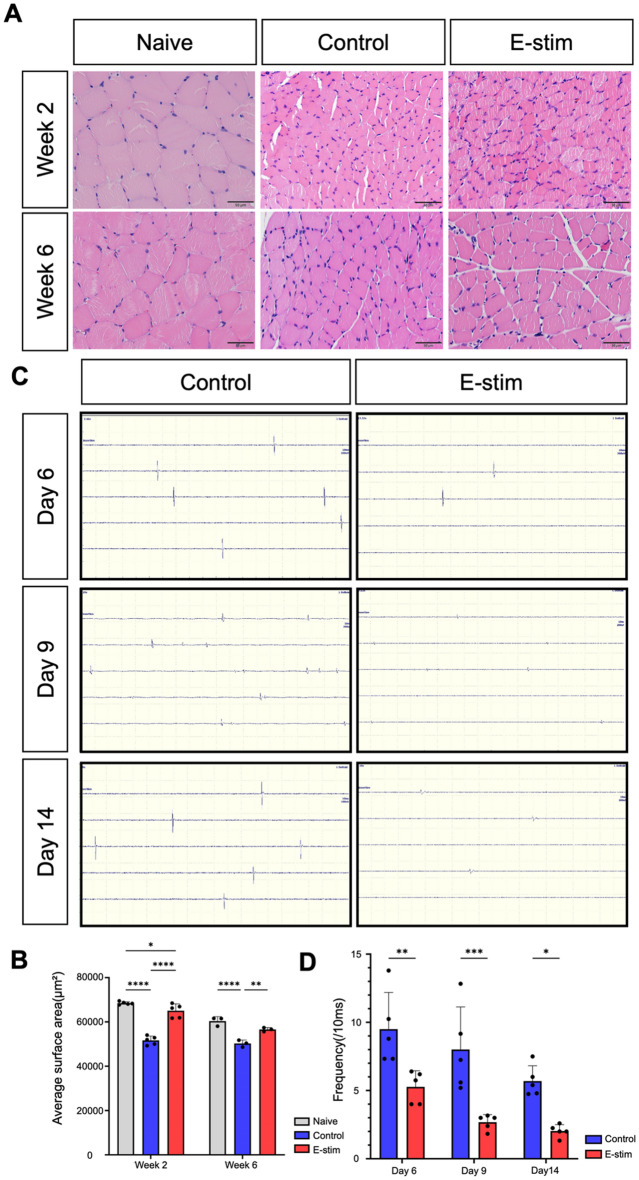
Distal electric stimulation ameliorates atrophy and abnormal autoelectricity of denervated muscle fiber. (**A**,** B**) Hematoxylin and Eosin staining of the cross-section of gastrocnemius muscle in each experimental group. The average surface area per fiber was calculated by three randomly picked myofiber under high power field of independent animal in each group at postoperative weeks 2 and 6. (*n* = 5, employing one-way ANOVA with Tukey multiple comparison analysis. * indicated *p* < 0.05; ** indicated *p* < 0.01, *** indicated *p* < 0.001, **** indicated *p* < 0.0001). (**C**,** D**) Electromyography recordings measured the frequency of muscle fibrillation in 100 micro second of five independent animals in each experimental group at postoperative days 6, 9, and 14 following nerve transection. The findings indicate a lower frequency of muscle fibrillation in the E-stim group compared to the Control group. (*n* = 5, employing t-tests analysis. * indicated *p* < 0.05; ** indicated *p* < 0.01, and *** indicated *p* < 0.001)

From the above information, we observed that the direct effect of distal E-stim started from week 2 post-injury, during the period when the injured muscle was still undergoing denervated atrophy (Fig. 1E). Therefore, we further investigated the influence of E-stim on the regulation of denervated muscle atrophy. Histological analysis of injured muscle tissue (Fig. 2A) was performed at weeks 2 and 6 post-injury. H&E staining revealed that muscle fibers in the E-stim group were more organized and exhibited less fiber contraction compared to the control group. Quantitative analysis of muscle fiber cross-sectional area (Fig. 2B) showed that the E-stim group had significantly larger muscle fibers than the control group at both time points (Week 2: E-stim: 65,075 ± 3,082 μm², Control: 51,660 ± 1,985 μm²; *p* < 0.0001; Week 6: E-stim: 56,583 ± 895.3 μm², Control: 50,288 ± 1,567 μm²; *p* < 0.01).

Fibrillation potential frequency in denervated muscle atrophy is a significant marker to assess the severity of muscle denervation [24, 25]. In this study, SFEMG (Fig. 2C) was conducted on days 6, 9, and 14 post-injury to assess the degree of muscle fiber denervation injury within 2 weeks. The E-stim group exhibited less frequent fibrillation compared to the control group, indicative of decreased spontaneous electromyographic activity in denervated muscle fibers. Quantitative analysis of nerve firing frequency (Fig. 2D) confirmed that the E-stim group had significantly lower frequencies at all measured time points (Day 6: E-stim: 5.3 ± 1.2 spikes/10 ms, Control: 9.5 ± 2.7 spikes/10 ms; *p* < 0.01; Day 9: E-stim: 2.7 ± 0.6 spikes/10 ms, Control: 8.0 ± 3.1 spikes/10 ms; *p* < 0.001; Day 14: E-stim: 2.0 ± 0.5 spikes/10 ms, Control: 5.7 ± 1.1 spikes/10 ms; *p* < 0.05). These findings reveal that the potential beneficial effects of distal E-stim may contribute to the alleviation of denervated muscle atrophy in the early stage of nerve injury.

### Preservation of neuromuscular junction homeostasis by distal nerve E-stim

**Fig. 3:**
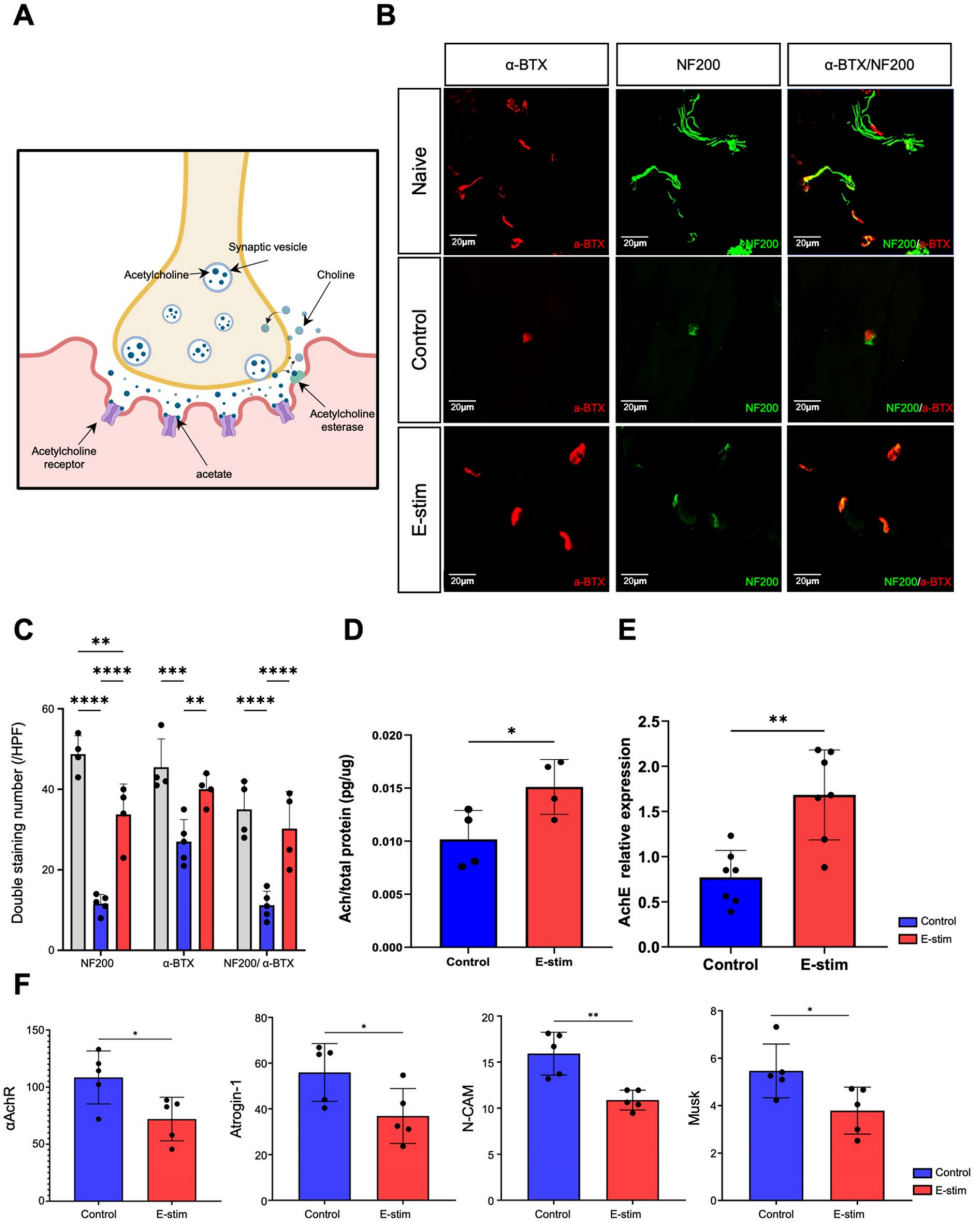
Histologic structure, neurotransmitter regulation and associated molecular regulation in neuromuscular junction. (**A**) Schematic illustration of the acetylcholine releasing at NMJ. (**B**,** C**) Immunofluorescent stain of the neuromuscular junction at week 1 post-injury in each experimental group, illustrating the density of double staining for pre- (NF200, green color) and postsynaptic (alpha-bungarotoxin, red color) markers each group. The accompanying bar chart on the right presents statistical indices of density and significance, revealing that the NMJ count in each experimental group. Scale bar = 50 μm. (**D**) Acetylcholine levels were quantified using ELISA array to assess ACh expression in muscle samples innervated by transected sciatic nerves in the Control and E-stim groups. The relative expression level is defined by the index normalized to the healthy contralateral side (right side) of the same rat. (**E**) Acetylcholinesterase expression level in the Control and E-stim groups. The expression level is normalized to the total protein expression of the sample. (**F**) Comparison of expression levels of neuron physical function-associated molecules between the E-stim and Control groups. (*n* = 5 per timepoint. Data was presented with mean standard deviation. Statistical analysis was conducted using Prism software, employing t-tests and one-way ANOVA with Tukey multiple comparison analysis. * indicated *p* < 0.05; ** indicated *p* < 0.01, *** indicated *p* < 0.001, **** indicated *p* < 0.0001)

We further explored the direct influence on neuromuscular junction of denervated muscle at week 1 post-injury. Comprehensive analysis of denervated NMJ provided insights into the underlying mechanisms regulating NMJ microenvironment with distal E-stim. ACh and AChE are crucial for the proper functioning and regeneration of NMJs [26, 27]. ACh acts as a neurotransmitter that triggers muscle contraction when released into the synaptic cleft. AChE, on the other hand, is responsible for the rapid breakdown of ACh, thus terminating the signal and allowing the muscle to relax (Fig. 3A). Immunofluorescent staining of NMJ components (Fig. 3B) showed increased colocalization of presynaptic NF200 and postsynaptic alpha-bungarotoxin (α-BTX) in the E-stim group, indicating better-preserved NMJ structure. Quantitative analysis (Fig. 3C) revealed higher double staining numbers per high-power field for NF200, α-BTX, and their co-localization in the E-stim group compared to controls (NF200: 33.8 ± 7.6 vs. 11.6 ± 2.3, *p* < 0.01; α-BTX: 40.0 ± 3.7 vs. 27.0 ± 5.5, *p* < 0.001; NF200/α-BTX: 30.3 ± 9.2 vs. 11.2 ± 3.5, *p* < 0.05). Biochemical assays (Fig. 3D) showed that ACh expression levels were significantly higher in the E-stim group compared to controls (0.015 ± 0.003 µg/g vs. 0.010 ± 0.003 µg/g; *p* < 0.05). Similarly, AChE protein levels (Fig. 3E) were elevated in the E-stim group (1.683 ± 0.499 vs. 0.770 ± 0.298; *p* < 0.01). The abovementioned elevated protein level of both ACh and AChE had demonstrated enhanced neurotransmitter and reuptake activity within NMJ. Additional analysis of gene regulation in denervated muscle (Fig. 3F) indicated significant downregulation in *α-AChR*,* Agrin-1*,* N-CAM*,* and MuSK* in the E-stim group compared to controls (α-AChR: 108.4 ± 23.21 vs. 72.01 ± 19.10, *p* < 0.05; Atrogin-1: 55.94 ± 12.65 vs. 36.88 ± 12.00, *p* < 0.05; N-CAM: 15.92 ± 2.33 vs. 10.88 ± 1.08, *p* < 0.01; MuSK: 5.46 ± 1.13 vs. 3.79 ± 0.99, *p* < 0.05), indicative of protective effect over denervated muscle atrophy.

### Regulation of satellite cell subpopulation in post-synaptic microenvironment

**Fig. 4:**
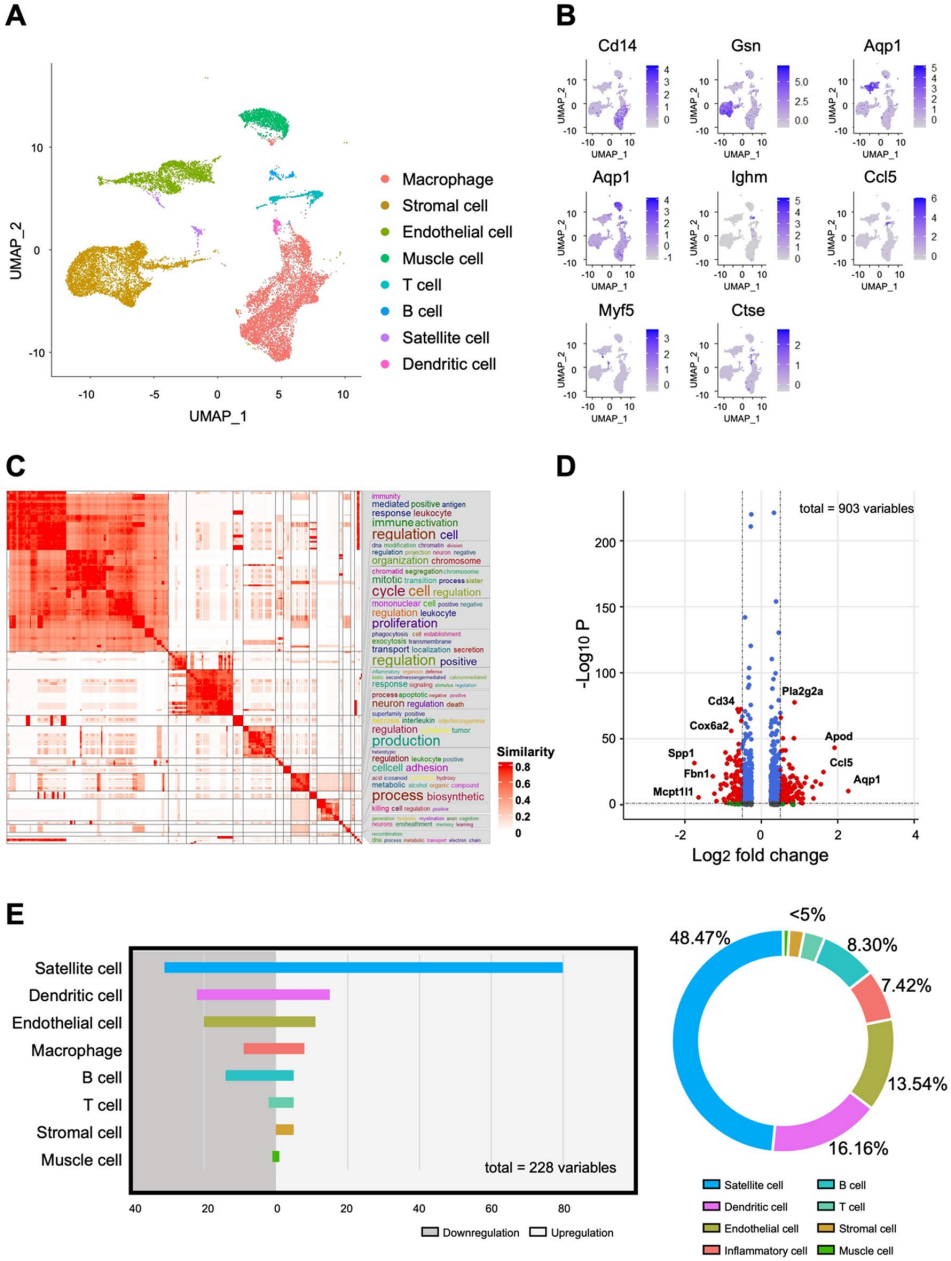
Single cell RNA sequencing in denervated muscle. (**A**) UMAP visualization conducted using the R package Seurat. Samples were mapped based on quality control criteria, including cells with nFeature RNA counts between 600 and 3000, while maintaining mitochondrial RNA levels below 40%. Three sample groups were merged: Naive (*n* = 2, total cell count = 11253), Control (*n* = 2, total cell count = 11780), and E-stim (*n* = 2, total cell count = 14280). (**B**) Feature plots of biomarkers for annotation. The color gradient represents the expression level of genes across the global UMAP, utilizing known housekeeping genes specific to different cell types. (**C**) Simplified GO enrichment heatmap processed using the R package simplifyEnrichment. The word cloud on the right side summarizes the frequency of functions modulated within the GO database. The size of characters represents keywords of functions, linearly correlated with the level of regulation. (**D**) Volcano plot illustrating all differentially expressed genes between the E-stim group and the Control group, plotted using the R package ggplot2 with screening criteria including a log2 fold change > 0.5d a p-value < 0.05. (**E**) Analysis of accumulated differentially expressed genes among each cluster, applying criteria of a log2 fold change > 0.5 and a p-value < 0.05. The highest count and percentage of differentially expressed genes is observed in the Satellite cell cluster, indicating that Satellite cells exhibit the most pronounced response to electrical stimulation

From the above findings, we identified that distal E-stim regulated the early NMJ microenvironment after denervation injury. However, the target cell population and the underlying mechanism have yet to be investigated. Therefore, we further investigated the regulatory mechanism within denervated muscles by utilizing scRNA-seq. RNA expression characteristics of cell clusters at postoperative day 3 were analyzed at single-cell resolution (Fig. 4A). Three sample groups were merged: Naive (*n* = 2, total cell count = 11253), Control (*n* = 2, total cell count = 11780), and E-stim (*n* = 2, total cell count = 14280) (Fig. S5). The UMAP plot demonstrated distinct clusters of cell populations, including muscle cells, Satellite cells, endothelial cells, stromal cells, and various immune cells. Feature plots of biomarkers for annotation (Fig. 4B) showed the expression levels of key genes across the global UMAP, utilizing known housekeeping genes specific to different cell types. The color gradient represented the expression level of genes, highlighting differential expression patterns among the cell populations. A simplified GO enrichment heatmap (Fig. 4C) was processed using the R package simplify Enrichment. This heatmap depicted the clustering of gene expression profiles, with significant enrichment in pathways related to immune function, cell cycle regulation, and muscle differentiation. The accompanying word cloud summarized the frequency of functions modulated within the GO database, with character size linearly correlated with the level of regulation. A volcano plot (Fig. 4D) illustrated all differentially expressed genes between the E-stim group and the Control group. Screening criteria included a log2 fold change > 0.5 and a p-value < 0.05. Notable upregulated genes in the E-stim group included *Cd34*,* Cox6a2*,* Spp1*,* and Apod*, while downregulated genes such as *Ccl5 and Mcpt1* highlighted the anti-inflammatory effects of E-stim. Analysis of accumulated differentially expressed genes among each cell cluster (Fig. 4E) applied criteria of a log2 fold change > 0.5 and a p-value < 0.05. The highest count and percentage of differentially expressed genes were observed in the Satellite cell cluster (48.47%), indicating that Satellite cells exhibited the most pronounced response to electrical stimulation. These findings underscore the critical role of Satellite cells in mediating the regenerative effects by distal E-stim.

### Myogenesis and angiogenesis are regulated in satellite cell population

**Fig. 5:**
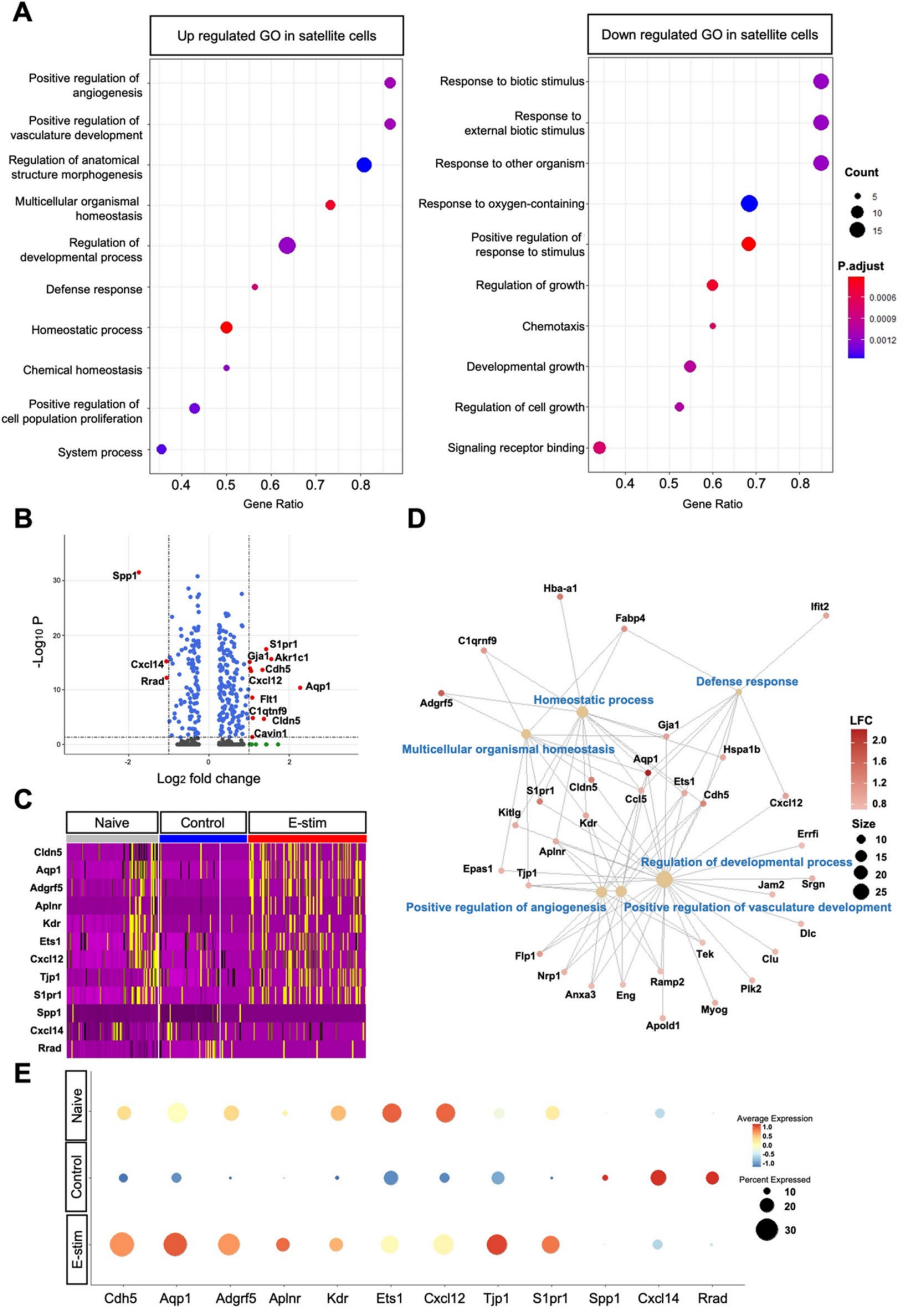
Gene ontology (GO) analysis of Satellite cell cluster by distal E-stim. (**A**) Dot plot depicting up and down-regulated GO terms within the E-stim Satellite cell cluster relative to their level in the Control Satellite cell. The size of the dots indicates the number of genes involved in a Gene Ontology (GO) category, while the color represents the significance of regulation. (**B**) Volcano plot illustrating the differential expression of genes between the E-stim Satellite cell and Control Satellite cell groups. Screening criteria included a log2 fold change > 1 and a p-value < 0.05. (**C**) Heatmap displaying Satellite cells across the Naive, Control, and E-stim groups. Genes were selected based on the criteria outlined in plot B. Genes highly modulated by electrical stimulation post-nerve transection are also prominently expressed in the Naive group, serving as a baseline for Satellite cell activity. (**D**) Network visualization depicting interactions among differentially expressed genes within the E-stim Satellite cell cluster. The size of the dots representing GO function names corresponds to the number of genes involved, while the color gradient of dots with gene codes indicates the log fold change of specific genes. (**E**) Dot plot illustrating the RNA expression level of highly modulated genes within the Satellite cell cluster. The size of the dots indicates the ratio of cells within the Satellite cell cluster expressing a particular gene. The color gradient represents the average expression level compared with the standardized gene expression across all cells within the Satellite cell population

Focusing on the Satellite cell cluster, the further GO analysis (Fig. 5A) revealed significant upregulation of GO terms related to positive regulation of angiogenesis, vasculature development, anatomical structure morphogenesis, multicellular organismal homeostasis, developmental processes, defense response, and chemical homeostasis in the E-stim group compared to controls. Conversely, downregulated GO terms included responses to biotic and external biotic stimuli, responses to other organisms, oxygen-containing compounds, chemotaxis, and regulation of growth and cell binding. A volcano plot (Fig. 5B) illustrated differential expression of genes between the E-stim and control Satellite cell groups. Notable genes such as *Aqp1*,* Cdh5*,* Cxcl12*,* Cldn5*,* S1pr1* were significantly upregulated, while *Spp1*,* Cxcl14*,* and Rrad* were significantly downregulated, indicating their potential role in the regulation of Satellite cell function by E-stim. Heatmap analysis (Fig. 5C) displayed Satellite cells across Naive, Control, and E-stim groups, with genes selected based on criteria outlined in the volcano plot. Genes highly modulated by E-stim postinjury were also prominently expressed in the Naive group, serving as a baseline for Satellite cell activity. Network visualization (Fig. 5D) depicted interactions among DEGs within the E-stim in the Satellite cell cluster. Key genes involved in homeostatic processes, multicellular organismal homeostasis, defense response, and positive regulation of developmental processes were prominently featured. A dot plot (Fig. 5E) illustrated the RNA expression level of highly modulated genes within the Satellite cell cluster. The size of the dots indicated the ratio of cells within the Satellite cell cluster expressing a particular gene, while the color gradient represented the average expression level compared to the standardized gene expression across all cells within the Satellite cell population.

### Validation of satellite cell differentiation and neovascularization by distal nerve E-stim

**Fig. 6:**
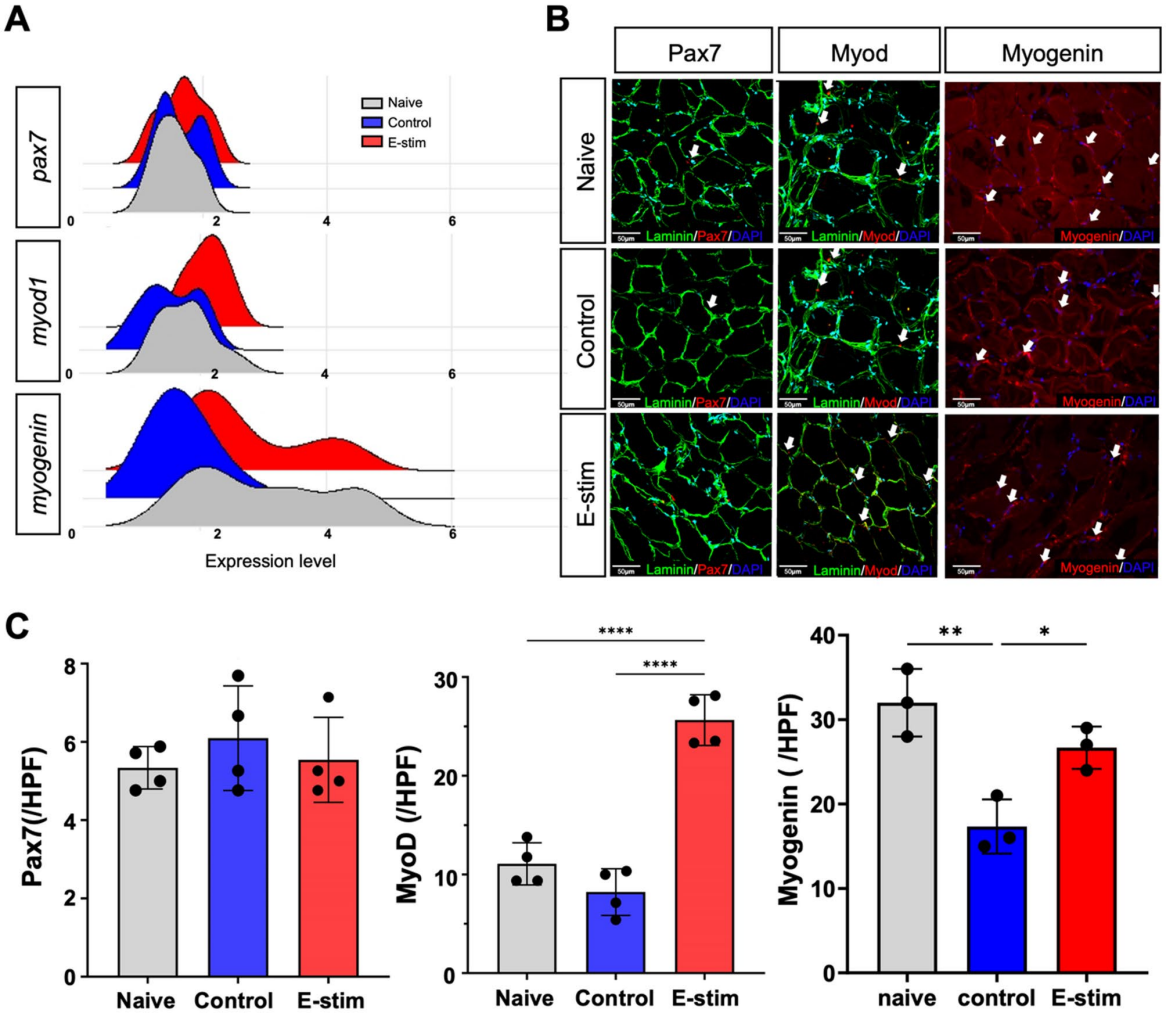
Satellite cell differentiation in denervated muscle by distal E-stim. (**A**) Ridge plot displaying the RNA expression levels of Pax7, MyoD, and Myogenin in Satellite cell clusters from different experimental groups three days post-nerve transection. Pax7 serves as a biomarker of quiescent Satellite cells, while activation of Satellite cells involves co-expression of Pax7 with MyoD. Myogenin is a transcription factor that regulates myocyte fusion during development. (**B**,** C**) Immunofluorescent staining of muscle tissue samples taken three days post-injury. The left column illustrates Pax7 distribution in myofibers (Red: Pax7/Green: Laminin/Blue: DAPI), with no significant differences noted in expression among the three groups. The middle column depicts MyoD-expressing cells in myofibers (Red: MyoD/Green: Laminin/Blue: DAPI), indicating activation of Satellite cells. The highest expression of MyoD is observed in the E-stim group. The column on the right displays myogenin-expressing cells in myofibers (Red: Myogenin/Blue: DAPI). The Naive group exhibits the highest level of myogenin expression, consistent with its unharmed sciatic nerve and intact neuromuscular function. Additionally, the E-stim group demonstrates a higher density of myogenin-expressing cells compared to the Control group. (*n* = 3–4 per group. Data was presented with mean standard deviation. Statistical analysis was conducted using Prism software, employing one-way ANOVA with Tukey multiple comparison analysis. * indicated *p* < 0.05; ** indicated *p* < 0.01, *** indicated *p* < 0.001, **** indicated *p* < 0.0001)

To further validate the role of Satellite cell in denervated muscle by E-stim, Gene expression analysis for monitoring the activation level of the Satellite cell cluster in Naive, Control, and E-stim groups was performed three days post-nerve injury (Fig. 6A). Ridge plots displayed RNA expression levels of Pax7, MyoD, and Myogenin in Satellite cell clusters. Pax7 served as a biomarker of quiescent Satellite cells, while activation of Satellite cells involved co-expression of Pax7 with MyoD. Myogenin, a transcription factor regulating myocyte fusion during development, showed higher expression levels in the E-stim group compared to controls. Immunofluorescent staining of muscle tissue samples (Fig. 6B) illustrated the distribution of Pax7, MyoD, and Myogenin in myofibers. The Pax7 expression showed no significant differences among the three groups (Naive: 5.3 ± 0.5, Control: 6.1 ± 1.3, E-stim: 5.5 ± 0.8; *p* > 0.05) (Fig. 6C). MyoD-expressing cells indicated activation of Satellite cells, with the highest expression observed in the E-stim group (Naive: 11.1 ± 2.1, Control: 8.2 ± 2.4, E-stim: 25.6 ± 2.6; *p* < 0.0001 (Fig. 6C). Myogenin-expressing cells were predominantly observed in the Naive group, reflecting the unharmed sciatic nerve and intact neuromuscular function. The E-stim group demonstrated a higher density of Myogenin-expressing cells compared to the Control group, suggesting enhanced muscle function (Naive: 32.0 ± 4.0, Control: 17.3 ± 3.2, E-stim: 26.7 ± 2.5; *p* < 0.05) (Fig. 6C). In brief, distal E-stim promote Satellite cell differentiation in denervated muscle injury. On top of the proteins mentioned above, S1pr1expression in E-stim group showed higher expressing level than the Control group (Fig. S6), implicating enhanced Satellite cell activation and progression [28, 29]. In addition, Apelin is known as a target of vascular niche with the potential to stimulate endogenous skeletal muscle repair [30], with a higher expression in the E-stim group than observed in the Control group (Naive: 109.03 ± 24.6, Control: 36.67 ± 26.5, E-stim: 106.7 ± 5.9; *p* < 0.05) (Fig. S7). Further investigation of neovascularization in the innervated muscle confirmed that CD31 and vWF had significantly higher expression in the E-stim group compared to the Control group (Naive: 25.33 ± 6.7, Control: 9.67 ± 3.2, E-stim: 22.33 ± 1.5; *p* < 0.05 for CD31; Naive: 15.67 ± 1.5, Control: 9.33 ± 1.5, E-stim: 15.02 ± 1.1; *p* < 0.01 for vWF) (Fig. S6).

## Discussion

This study shows that distal E-stim significantly enhances neuromuscular reinnervation and functional recovery after peripheral nerve injury. E-stim improved functional gait scores, increased muscle mass, and reduced muscle atrophy by alleviating muscle fiber atrophy and promoting regeneration. Enhanced NMJ preservation was indicated by increased colocalization of presynaptic NF200 and postsynaptic α-BTX, along with elevated acetylcholine and acetylcholinesterase levels. scRNA-seq revealed that E-stim modulates Satellite cell subpopulations, upregulating genes associated with angiogenesis and muscle differentiation, fostering a regenerative environment. Our findings indicate that E-stim promotes both axon regeneration and muscle recovery. These results have significant implications for developing effective therapies for peripheral nerve injuries.

It is important to note that our previous research [21] confirmed that E-stim to the distal stump of an injured nerve facilitated neuromuscular regeneration and functional recovery through (1) preservation of muscle mass; (2) improved functional recovery; (3) enhanced neuromuscular junctions; and (4) electrophysiological benefits. To our knowledge, this is the first research to reveal the distinct beneficial effect of distal E-stim on NMJ regeneration. In the present research, we further investigated the underlying regulatory mechanisms. To simplify the experimental condition, we use single-session stimulation to explore the beneficial mechanisms. Although not optimal for a single session, we did observe a significant benefit on functional outcomes from our previous findings [21]. A similar concept of single proximal E-stim has also been approved effective in promoting peripheral nerve regeneration by Tessa Gordon’s group [16–18, 20, 31]. The use of single distal E-stim has been shown to preserve the integrity of the NMJ and reduce muscle atrophy. Immunofluorescent staining revealed a reduction in single muscle fibrillation (Fig. 2B), better-preserved NMJ structures (Fig. 3A), and higher levels of neurotransmitter activity (Fig. 3D, E) in the E-stim group. These findings suggest that distal E-stim facilitates the maintenance of NMJ homeostasis, which is essential for efficient neuromuscular transmission and muscle contraction. This beneficial effect differs from the proximal E-stim proposed by Tessa Gordon [18, 31], which primarily targets the neuronal cell body and is characterized by elevated cyclic adenosine monophosphate levels and increased expression of neurotrophic factors and other growth-associated genes, including cytoskeletal proteins. In contrast, our proposed distal E-stim did not involve in regulating axon sprouting and neuroinflammation at the proximal nerve stump at week 6 (Fig. S2-S4).

ACh plays a crucial role in NMJ regulation by acting as the primary neurotransmitter responsible for muscle contraction [32]. AChE is essential in controlling the duration of ACh action at cholinergic synapses, ensuring the timely termination of its effects on receptors [26]. The high density of muscle-type Ach receptors in the synaptic cleft, coupled with the excess release of ACh under normal conditions, contributes to the reliability of NMJs [27]. Studies show that disorganized NMJs can result from the regeneration process, highlighting the importance of well-regulated ACh and AChE activities [33]. The extracellular matrix at NMJs, rich in AChE, plays a role in directing synapse differentiation during regeneration [34]. Further, precise localization of AChE in the synaptic cleft is essential for efficient hydrolysis of ACh, ensuring proper neuromuscular transmission and preventing detrimental reattachment of ACh [27]. In this study, we observed significant regulation of neurotransmitter activity at the NMJ through the expression of ACh and AChE. The levels of ACh were notably higher in the distal E-stim group compared to controls (Fig. 3D), indicating enhanced synaptic transmission. Additionally, AChE levels were elevated in the E-stim group (Fig. 3E), suggesting efficient breakdown and reuptake of ACh. This regulation is critical for preventing continuous muscle stimulation and ensuring proper muscle relaxation between contractions. The increased expression of both ACh and AChE implies that distal E-stim not only enhances neurotransmitter release but also maintains neurotransmitter homeostasis at the NMJ. This balance is crucial for NMJ regeneration and maintenance, as it ensures efficient neuromuscular transmission and muscle contraction, ultimately contributing to better functional recovery and muscle health.

To investigate how distal E-stim regulates NMJ homeostasis and reinnervation, we used scRNA-seq to examine affected cell populations and pathways. This technique has enhanced the understanding of skeletal muscle regeneration by revealing cellular heterogeneity and transcriptional signatures [35]. Research indicates that muscle regeneration involves interactions between myogenic and non-myogenic cells, with repair mechanisms linked to changes in myogenic stem/progenitor cell states [36]. The regenerative capacity of skeletal muscle depends on muscle stem cell and their interactions with various cell types, underscoring the need to study cellular composition and heterogeneity in muscle tissues [37, 38]. In our study, key genes such as Pax7, MyoD, and Myogenin showed differential expression patterns, with MyoD and Myogenin being upregulated in the E-stim group, indicating enhanced Satellite cell activation and differentiation (Fig. 6). Additionally, genes involved in angiogenesis (Cd34) and anti-inflammatory responses (Apod) were also upregulated, suggesting a conducive environment for muscle repair and regeneration (Fig. 5). Our study provides a comprehensive analysis of MuSC dynamics and metabolic regulation during regeneration, leveraging scRNA-seq and pseudotemporal analysis. By comparing our findings with existing studies, we validate and extend the current understanding of MuSC behavior. The study by Dell’Orso et al. [39] identified distinct clusters of MuSCs and emphasized transcriptional heterogeneity and metabolic regulation, which aligns with our findings. Our integration of pseudotemporal analysis offers a dynamic view of MuSC transitions from quiescence to activation, enhancing the understanding of their regenerative capabilities. Similarly, Andrea et al. [40] presented a detailed single-cell transcriptomic atlas of muscle regeneration, identifying hierarchical relationships and paracrine signaling factors. Our study extends these findings by focusing on specific metabolic pathways and their connectivity during MuSC activation and differentiation. Moreover, our research, together with the findings of Barruet et al. [41], highlights the crucial role of understanding Satellite cell progression and differentiation in developing targeted therapies for muscle regeneration. The consistent identification of distinct subpopulations across different studies suggests a stable and reproducible Satellite cell hierarchy that is crucial for maintaining muscle homeostasis and regeneration. The combined insights from these studies highlight the potential for refining therapeutic strategies by targeting specific Satellite cell subpopulations to enhance regenerative outcomes.

Satellite cells, tissue-specific stem cells in skeletal muscle, play a crucial role in muscle regeneration and fiber differentiation. These cells are responsible for maintaining the regenerative capacity of skeletal muscle [42, 43]. Satellite cells undergo activation, proliferation, and differentiation processes to facilitate muscle regeneration, which is essential for repairing myofibers damaged during exercise or injury [44, 45]. They exhibit a high degree of population heterogeneity and are regulated by intrinsic and extrinsic mechanisms, including signals from the niche and communication with various stromal cell types in their microenvironment [46]. In terms of skeletal muscle regeneration and differentiation, Pax7, MyoD, and Myogenin play crucial roles in the regulation of Satellite cells [47]. Pax7 is a marker for Satellite cells and is essential for their maintenance and activation [48, 49]. MyoD is a myogenic regulatory factor that plays a key role in the determination and differentiation of myoblasts into myocytes, contributing to muscle repair and regeneration [48]. Additionally, Myogenin, another myogenic regulatory factor, is involved in the terminal differentiation and fusion of myoblasts into mature muscle fibers, further aiding in skeletal muscle regeneration [50]. These proteins orchestrate the intricate process of Satellite cell activation, proliferation, and differentiation, ensuring efficient muscle repair and regeneration in response to injury or exercise-induced damage. In this study, Pax7 expression in the denervated muscle showed no significant differences in expression among the Naive, Control, and E-stim groups (Fig. 6). However, the expression of MyoD, which is indicative of activated Satellite cells, was significantly higher in the E-stim group compared to controls (Fig. 6), suggesting that distal E-stim promotes Satellite cell activation. Additionally, MyoG exhibited elevated expression in the E-stim group (Fig. 6). These findings indicate that distal E-stim enhances Satellite cells differentiation into mature muscle cells. The upregulation of MyoD and Myogenin implies that E-stim facilitates the regenerative process of Satellite cells, contributing to muscle repair and regeneration. This enhanced Satellite cell activity is crucial for effective neuromuscular recovery, as it ensures the replenishment and differentiation of muscle fibers following injury. Certain diseases, such as statin-induced muscle wasting, are characterized by symptoms that range from mild myalgia to severe rhabdomyolysis following the administration of cholesterol-lowering drugs. A key underlying mechanism in statin-induced muscle wasting is impaired muscle regeneration, which affects the proliferation and differentiation of satellite cells, including the expression of MyoD [51]. From this perspective, the translational value of strategies to prevent statin-induced muscle wasting should be considered, especially for high-risk patient groups, including older adults, women, individuals of Asian descent, and those with comorbidities such as diabetes or renal impairment [52].

While this study provides valuable insights into the benefits of E-stim, it is important to acknowledge its limitations. We only presented the major molecular regulation within denervated muscle by distal E-stim three days post-treatment. Further studies with extended follow-up periods are necessary to confirm the long-term benefits and comprehensive modulation of this treatment. Additionally, more research is needed to elucidate the precise independent molecular mechanisms through which E-stim modulates Satellite cell function and NMJ regeneration. Moreover, analyzing multinucleated muscle cells using scRNA-seq poses challenges due to their large, multinucleated nature, making them resistant to traditional single-cell techniques. Williams et al. [38] discuss the challenges of applying scRNA-seq to large multinucleated cells like myofibers and highlight the use of single-nucleus RNA-seq as a complementary method. They found that nuclear heterogeneity within myofibers plays a crucial role in muscle function and disease, with specific nuclei expressing genes essential for NMJ function. A follow-up validation analysis using snRNA-seq to confirm these findings is essential. The positive outcomes observed with distal E-stim suggest its potential as a supplementary therapeutic approach for patients with peripheral nerve injuries. Future studies should aim to validate these findings in larger cohorts and investigate the long-term safety and efficacy of E-stim. A comprehensive understanding of the molecular mechanisms underlying E-stim’s effects, partly revealed by our research, will be crucial for optimizing treatment protocols and maximizing clinical benefits. This study significantly contributes to the field by pioneering the use of distal E-stim for enhancing neuromuscular reinnervation and functional recovery, thus offering new insights and paving the way for advanced therapeutic strategies in the treatment of peripheral nerve injuries.

## Conclusion

This study shows that distal E-stim significantly enhances neuromuscular reinnervation and functional recovery after peripheral nerve injury. E-stim improved functional gait scores, increased muscle mass, and reduced muscle atrophy by alleviating muscle fiber atrophy and promoting regeneration. Enhanced NMJ preservation was indicated by increased colocalization of presynaptic NF200 and postsynaptic α-BTX, along with elevated acetylcholine and acetylcholinesterase levels. scRNA-seq revealed that E-stim modulates Satellite cell subpopulations, upregulating genes associated with angiogenesis and muscle differentiation, fostering a regenerative environment. Our findings indicate that E-stim promotes both axon regeneration and muscle recovery. These results have significant implications for developing effective therapies for peripheral nerve injuries.

## Electronic supplementary material

Below is the link to the electronic supplementary material.

Supplementary Material 1

## Data Availability

Sequencing data generated or analyzed during this study are included in this published article and its Supplementary Information files. RNA-seq data generated in the study can be accessed at the Gene Expression Omnibus under accession code GSE281936. (https://www.ncbi.nlm.nih.gov/geo/query/acc.cgi?acc=GSE281936)

## Abbreviations

α-BTX: Alpha-bungarotoxin
ACh: Acetylcholine
AChE: Acetylcholinesterase
DEGs: Differentially expressed genes
E-stim: Electrical stimulation
GAPDH: Glyceraldehyde 3-phosphate dehydrogenase
GO: Gene ontology
H&E: Hemotoxylin and eosin
IT: Intermediary toe spread
NF200: Neurofilament 200
NMJ: Neuromuscular junction
PL: Print length
scRNA-seq: single-cell RNA sequence
SFEMG: Single fiber electromyography
SFI: Sciatic function index
TS: Toe spread
