# Supplementary material for: Distal electrical stimulation enhances neuromuscular reinnervation and satellite cell differentiation for functional recovery
Source: Stem Cell Res Ther. 2025 Jun 23;16:322. doi: 10.1186/s13287-025-04459-3 (PMC12186361; doi:10.1186/s13287-025-04459-3)
Supplement: Supplementary file 1 — Supplementary Material 1 [file 13287_2025_4459_MOESM1_ESM.docx]

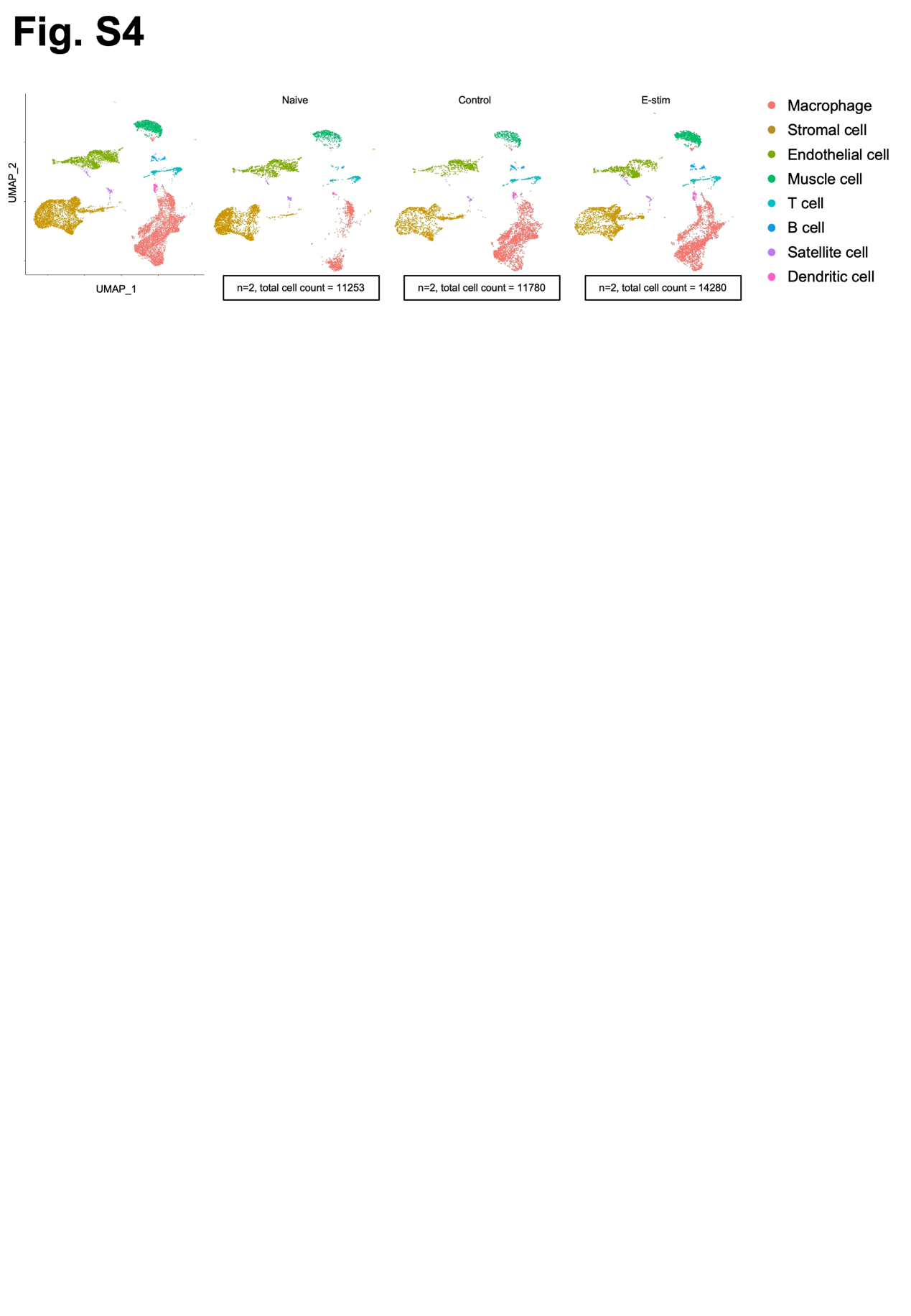


**Fig. S1. Dimensionality Reduction Plot (DimPlot) splited by Condition (Naive, Control, E-stim).** The DimPlot visualizes the clustering of single cells in a reduced dimensional space, with cells splited by experimental condition: Naive, Control, and E-stim. Each dot represents an individual cell, and the spatial arrangement reflects similarities in gene expression profiles. The distinct clustering of cells from each condition indicates biological variation. For each experimental condition, the sample size is two (n = 2). In Naïve, Control, and E-stim group, the total cell count are 11253, 11780, 14280 respectively.

**
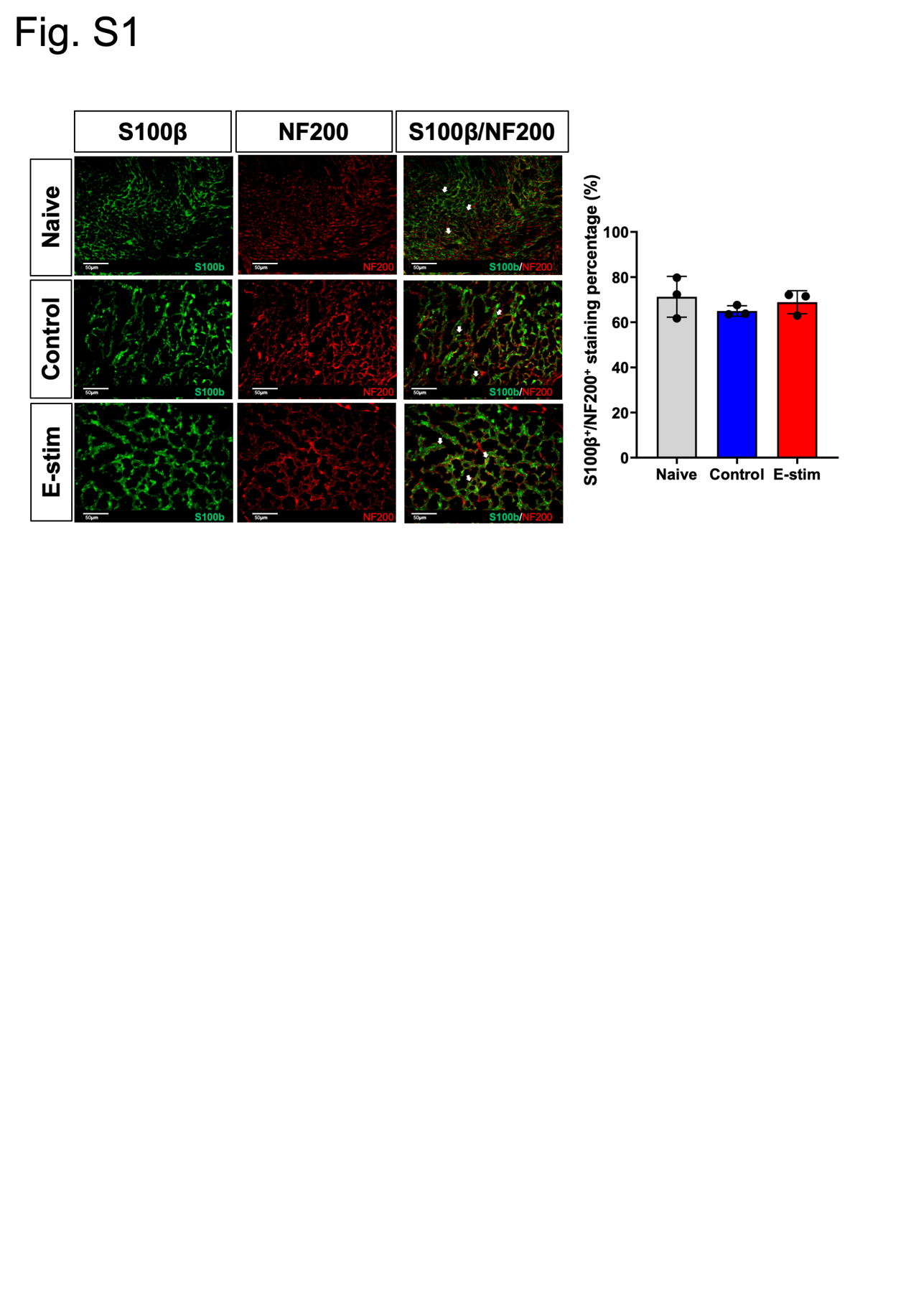
**

**Fig. S2. Immunofluorescent staining of S100β and NF200 in proximal nerve stump.** Left Panel: Representative images of S100β (green) and NF200 (red) staining in proximal nerve stump from naive, control, and E-stim groups at week 6. Scale bar = 50 µm. Right Panel: Quantitative analysis of the percentage of S100β^+^ and NF200^+^ staining cells. No statistical difference of S100β^+^/NF200^+^ cells was found in E-stim group as compared to the other two groups. (n = 3 per group. Data are presented as mean ± SD).


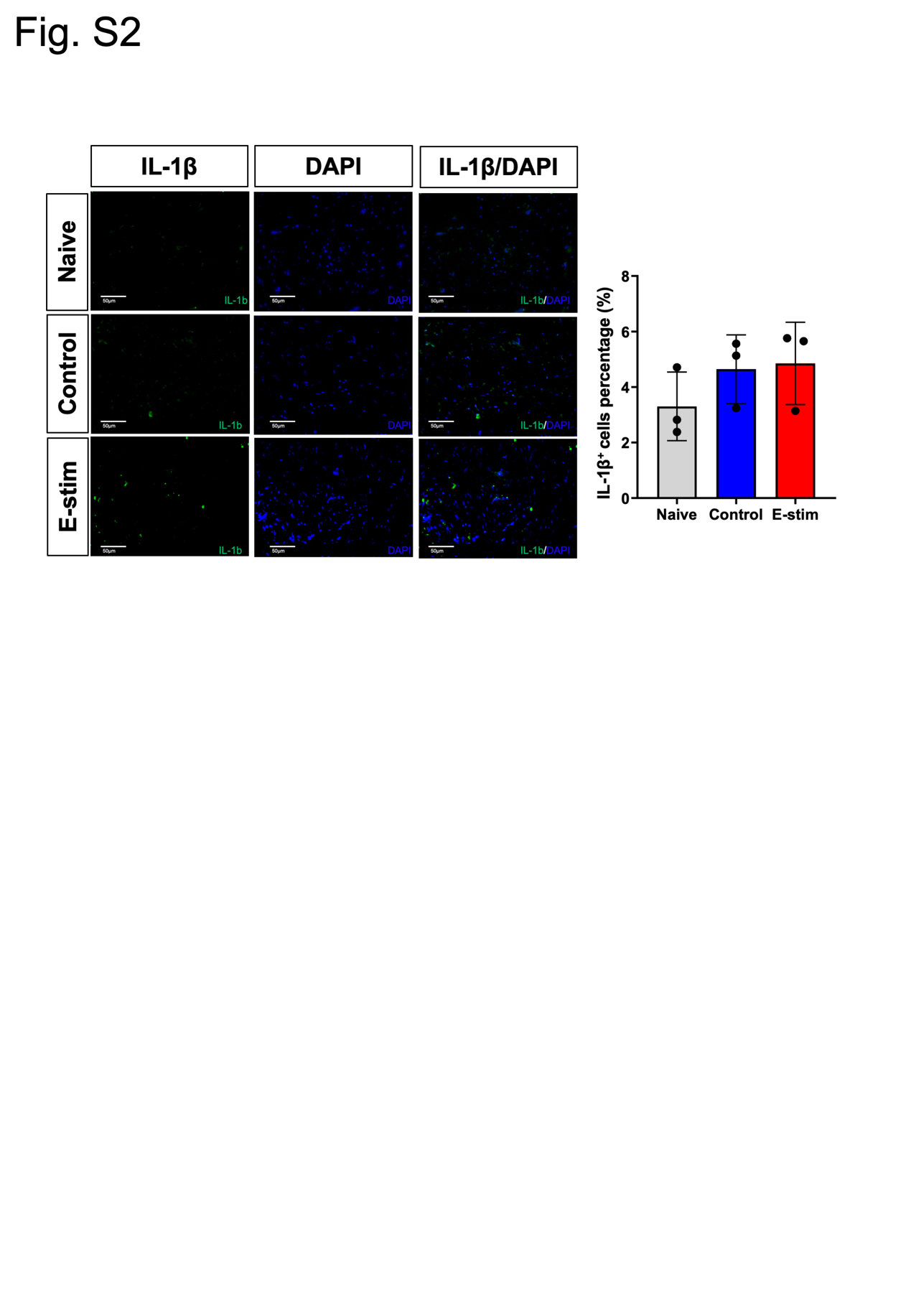


**Fig. S3. Immunofluorescent staining of IL-1β in proximal nerve stump.** Left Panel: Representative images of IL-1β (green) and DAPI (blue) staining in proximal nerve stump from naive, control, and E-stim groups at week 6. Scale bar = 50 µm. Right Panel: Quantitative analysis of IL-1β^+^ cells per total cell count. No statistical difference of IL-1β^+^ cells was found in E-stim group as compared to the other two groups. (n = 3 per group. Data are presented as mean ± SD).


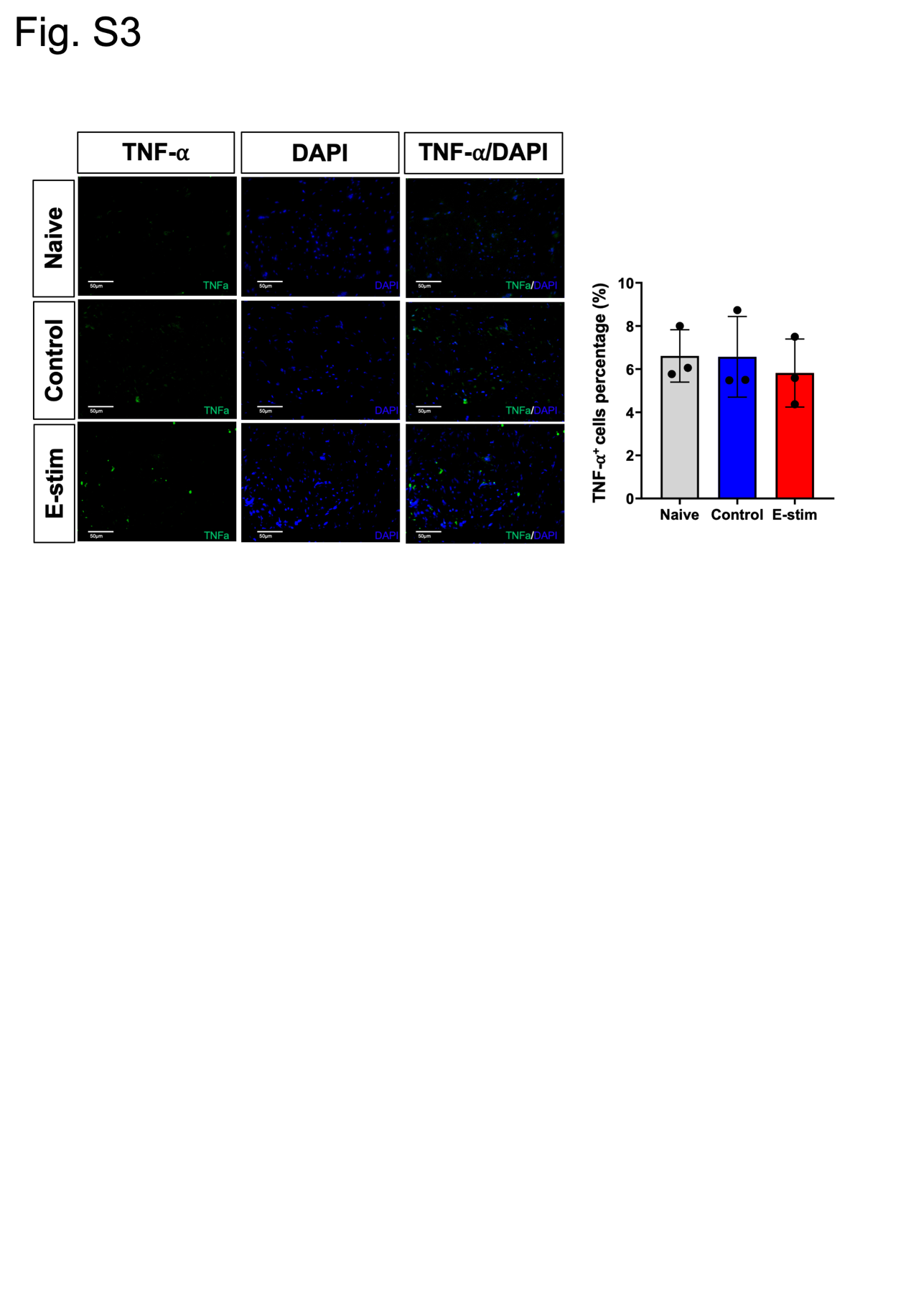


**Fig. S4. Immunofluorescent staining of TNF-α in proximal nerve stump.** Left Panel: Representative images of TNF-α (green) and DAPI (blue) staining in proximal nerve stump from naive, control, and E-stim groups at week 6. Scale bar = 50 µm. Right Panel: Quantitative analysis of TNF-α^+^ cells per total cell count. No statistical difference of TNF-α^+^ cells was found in E-stim group as compared to the other two groups. (n = 3 per group. Data are presented as mean ± SD).


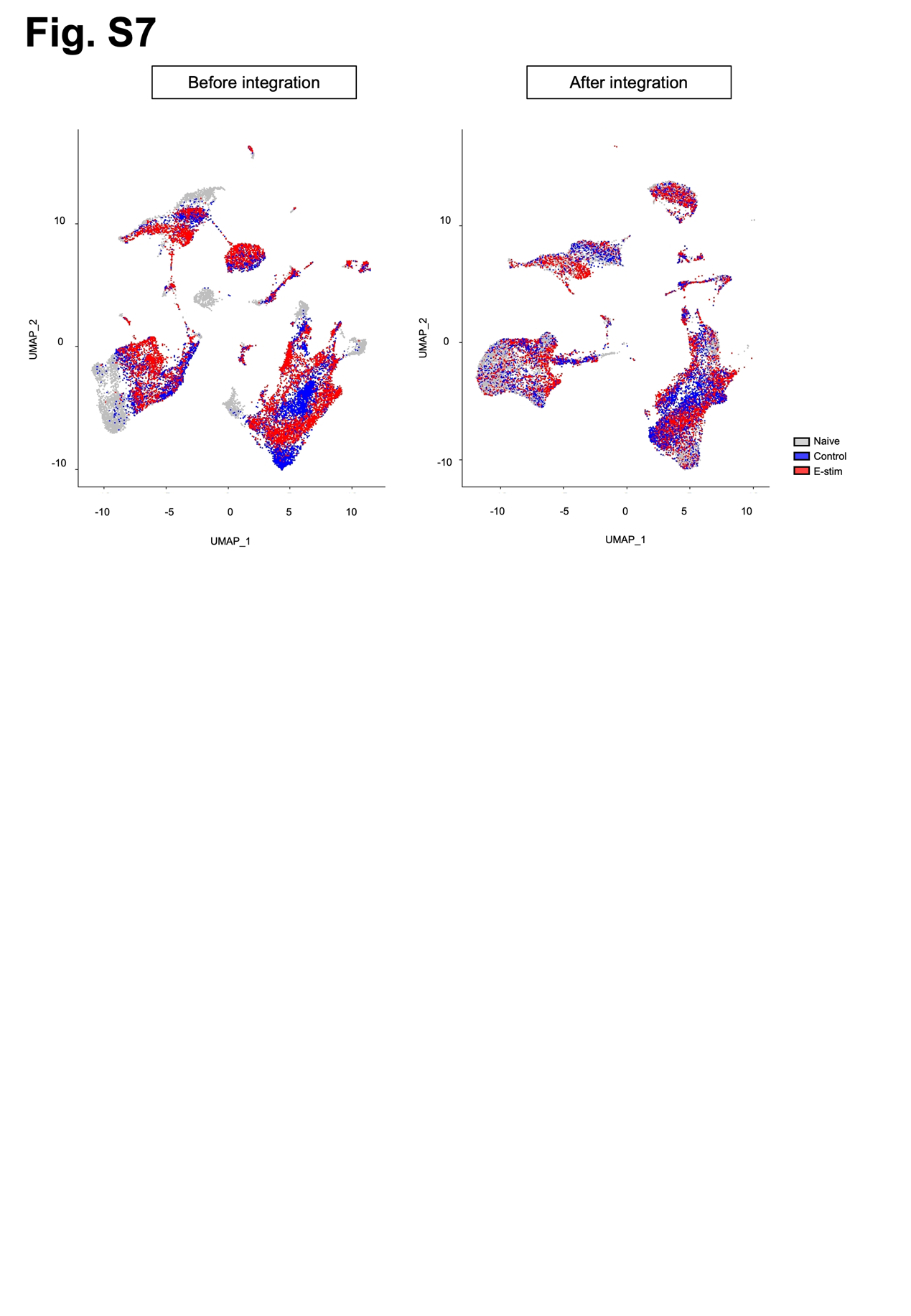


**Fig. S5. Correction of Batch Effects in Single-Cell RNA Sequencing Data.** This figure demonstrates the correction of batch effects in single-cell RNA sequencing data using integration techniques. Left Panel (Before integration): The UMAP plot shows the distribution of cells from different batches prior to integration. Each dot represents a single cell, colored by batch. The presence of distinct clusters of cells from different batches indicates significant batch effects, which can obscure biological signals. Right Panel (After integration): The UMAP plot displays the same cells after applying an integration method to correct for batch effects. Post-integration, cells from different batches (represented by different colors) are more intermixed, indicating successful batch effect correction. This integration ensures that observed variations are more likely to reflect true biological differences rather than technical artifacts.


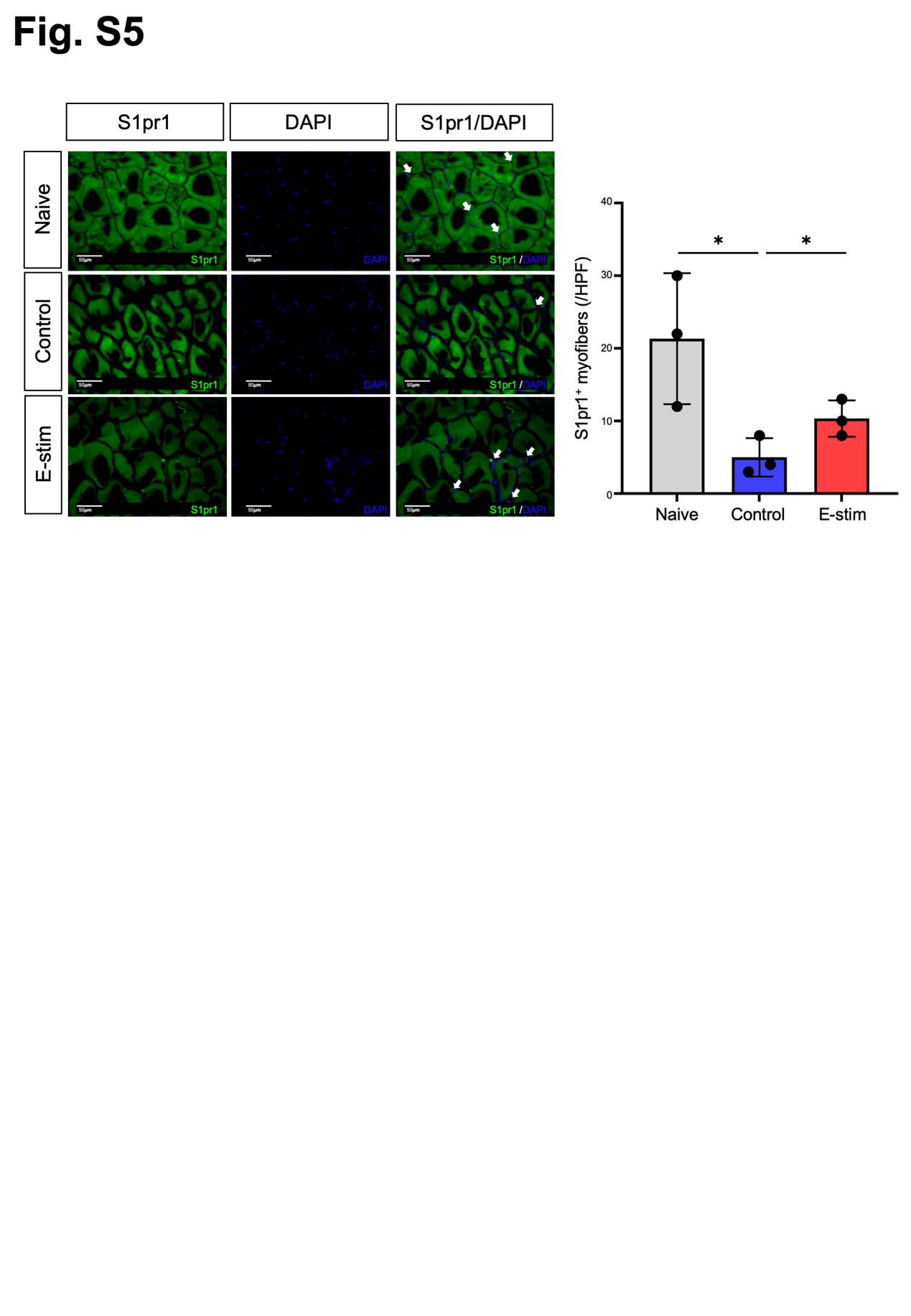


**Fig. S6. Immunofluorescent staining of S1pr1 in skeleton muscle.** Left Panel: Representative images of S1pr1 (green) and DAPI (blue) staining in skeleton muscle from naive, control, and E-stim groups at week 6. Scale bar = 50 µm. Right Panel: Quantitative analysis of the percentage of S1pr1 and DAPI staining cells. Statistical difference of S1pr1^+^/DAPI ^+^ myofiber was found in E-stim group as compared to the Control group. (n = 3 per group. Data are presented as mean ± SD, statistical significance is denoted as follows: *p < 0.05).


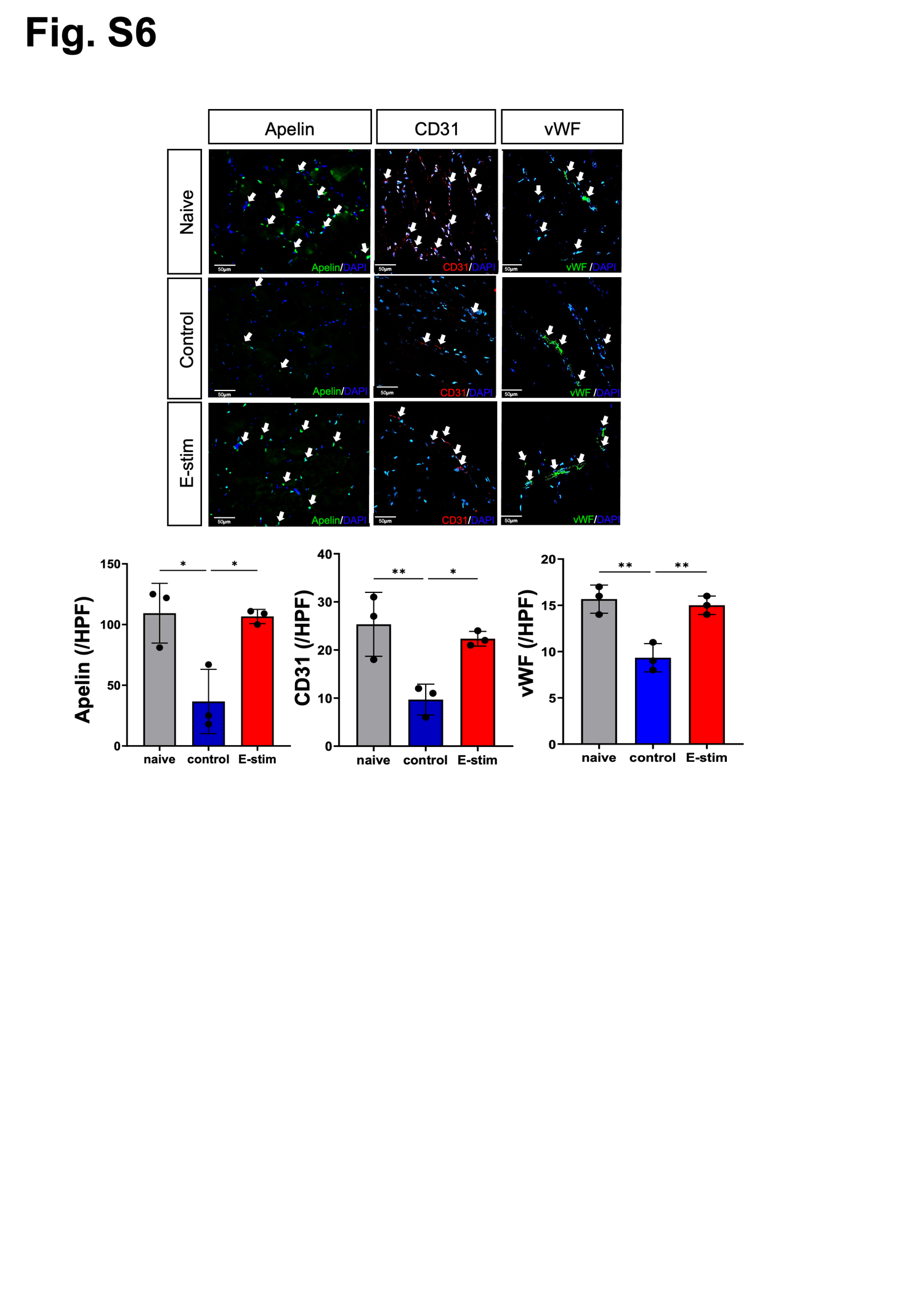


**Fig. S7. Immunofluorescent staining of angiogenesis in skeleton muscle.** Upper Panel: Representative images of Apelin (green), CD31 (red), vWF (green) and DAPI (blue) staining in skeleton muscle from naive, control, and E-stim groups at week 6. Scale bar = 50 µm. Lower Panel: Quantitative analysis of the percentage of Apelin/CD31/vWF staining cells. Statistical difference of Apelin^+^/CD31^+^/vWF^+^ myofibers was found in E-stim group as compared to the Control group. (n = 3 per group. Data are presented as mean ± SD, statistical significance is denoted as follows: *p < 0.05, **p < 0.01).
